# Emergency-Care Utilisation During Two Healthcare Crises in South Korea: A Single-Centre Comparison of Calendar-Aligned 32-Week Windows

**DOI:** 10.3390/jcm15207783

**Published:** 2026-10-09

**Authors:** Chan Park, Kang Hyun Lee, Oh Hyun Kim, Sun Ju Kim

**Affiliations:** Department of Emergency Medicine, Wonju Severance Christian Hospital, Yonsei University Wonju College of Medicine, Wonju 26426, Republic of Korea; qkrcks11@naver.com (C.P.); ed119@yonsei.ac.kr (K.H.L.); ohkim@yonsei.ac.kr (O.H.K.)

**Keywords:** emergency care, healthcare workforce, COVID-19, interhospital transfer, intensive care, triage

## Abstract

**Background/Objectives:** Emergency department (ED) attendance may change differently from subsequent hospital utilisation during healthcare disruptions. We compared two historical crisis episodes within one Korean regional emergency medical centre. **Methods:** Four calendar-aligned 224-day windows before and after the onset of COVID-19 and the 2024 workforce disruption were analysed using episode-by-phase interactions. Modified Poisson models used patient-and-day two-way clustered variance. Less-conditioned and initial-acuity-conditioned models were compared. Daily counts used calendar-lag autocorrelation-consistent variance. Source-data audits, full-period segmented analyses and alternative-baseline sensitivities were added. **Results:** The windows included 83,998 visits by 66,096 patients after source-data recoding. The ratio of relative changes was 0.73 (95% confidence interval [CI], 0.68–0.78) for daily visits, 1.28 (1.19–1.37) for transfer-in presentation and 1.44 (1.33–1.55) for acuity-conditioned ICU admission. EMS arrival increased more during COVID-19, but the corrected physiological composite showed no clear between-episode contrast. ED-origin ICU admissions/day were 9.24 before and 9.67 during the workforce episode; the primary ratio of incidence rate ratios was 1.13 (1.05–1.22), but the prior-calendar-year sensitivity included the null (1.06, 0.97–1.15). Workforce-period mortality associations differed before and after acuity conditioning, with adjusted risk ratios of 1.28 (1.10–1.49) and 1.06 (0.92–1.22), respectively. **Conclusions:** Attendance contracted more than ED-origin ICU admission volume during the observed workforce episode. These findings describe utilisation, not measured capacity pressure, and depend partly on baseline and adjustment choices. The contribution is a multidimensional approach to utilisation surveillance, not universal crisis-specific effects or evidence of unchanged survival.

## 1. Introduction

Emergency care includes arrival, triage, interhospital referral and subsequent hospital treatment. The input–throughput–output framework explains why ED attendance alone may not represent the composition of care delivered or downstream hospital utilisation [1]. A reduction in visits can coexist with a higher proportion of patients admitted, without establishing either greater absolute workload or lower operational pressure.

Reductions in attendance during infectious outbreaks are well described [2,3,4]. National Korean data documented differential changes across acuity and emergency-institution levels during early COVID-19 [2]. More recent studies have examined the 2024 workforce disruption through critical-care utilisation, ED attendance, hospital outcomes and transfers [5,6,7,8,9]. Earlier strike studies and systematic reviews describe heterogeneous operational and mortality findings [10,11,12]; staffing shortages outside formal industrial action can also affect service delivery [13]. These observations motivate measurement across several stages, but do not establish that infectious and workforce crises have universally distinct effects.

We compared the preceding and early phases of two crises within the same regional emergency medical centre using equal-duration, calendar-aligned windows. The comparative estimand was the ratio of their within-episode relative changes. The episodes occurred in different historical settings; calendar alignment was not intended to make their baseline populations exchangeable. Full-period data were used for contextual and supplementary longitudinal analyses. Diagnostic composition was assessed as clinical context rather than a substitute for acuity.

## 2. Materials and Methods

### 2.1. Setting, Data Sources and Ethics

We performed a retrospective, single-centre comparison at Wonju Severance Christian Hospital, a tertiary regional emergency medical centre in South Korea. ED, resuscitation, discharge, ICU and inpatient records from 1 January 2017 to 30 September 2024 were linked using source encounter identifiers. All registered ED encounters were eligible; repeat visits remained separate observations. Patient identifiers were used only for linkage and clustered variance estimation in the analysis environment. Reporting followed RECORD and STROBE [14,15].

The Institutional Review Board of Wonju Severance Christian Hospital, Yonsei University Wonju College of Medicine, approved the study (CR 324132; 18 February 2025) and waived study-specific informed consent. Only hospital-recorded encounters and linked admissions were evaluated. Separate enquiry and pre-registration screening datasets were not incorporated.

### 2.2. Historical Episodes and Institutional Context

The primary analysis included four separate 224-day windows: 14 July 2019–22 February 2020 and 23 February–3 October 2020 for the COVID-19 episode; 11 July 2023–19 February 2024 and 20 February–30 September 2024 for the workforce episode. Thus, 896 analytical days were distributed across four windows, not a continuous observation period. COVID-19 onset and the contextual recovery transition on 1 June 2023 corresponded to national alert changes [16,17]. The workforce window covered only the initial 224 days of the 2024 disruption.

The exposure was a calendar-defined period, not a measured staffing decrement. Administrative resignation dates could not reliably identify actual clinical withdrawal. Service-availability reports also did not represent stable daily capacity. No dated institutional policy or operational announcement was available to define a local intervention. The investigators reported informal low-acuity redirection before registration in the context of nationwide messaging, but its timing, reach and consistency could not be quantified. These contextual observations were not coded as measured exposures or used to reconstruct unavailable staffing.

National measures accompanied the disruption. The February 2024 response included advice directing lower-acuity patients towards local facilities and support for severe/emergency care and transfer coordination [18]. A higher patient cost-sharing requirement for lower-acuity presentations at designated emergency centres took effect on 13 September 2024 [19]. These contemporaneous changes could have influenced attendance and selection, and cannot be disentangled from workforce disruption in this design.

### 2.3. Source-Data Audit and Variable Definitions

A revision-stage source audit identified two consequential discrepancies in the submitted analysis. First, 1219 infant ages recorded in months were missing during numeric conversion. They were recovered as months divided by 12, without imputation. Second, the archived physiological indicator combined blood pressure, heart rate, respiratory rate and GCS but omitted the temperature component described in the submitted Methods. All affected models were rerun using the stated five-component definition; the earlier four-component implementation was retained only as a clearly labelled sensitivity analysis. Table S14 documents these corrections.

The revised composite indicated at least one valid arrival measurement meeting systolic blood pressure < 90 mmHg, heart rate > 120/min, respiratory rate < 10 or >30/min, temperature < 35 °C or ≥38 °C, or Glasgow Coma Scale (GCS) ≤ 13. Sentinel and implausible values were removed using the explicit rules in Supplementary Methods S1. If all five components were unavailable, the composite was missing. If some were recorded and none was abnormal, this denoted no recorded abnormality, not confirmation that every component was normal. Completeness-stratified and stricter-definition sensitivities addressed this distinction. Historical measurement protocols could not be independently verified; component-specific recording and abnormality rates were compared across windows.

Front-door outcomes were recorded for transfer-in presentation, 119 emergency medical service (EMS) arrival, initial Korean Triage and Acuity Scale (KTAS) levels 1–2, and the recorded physiological-abnormality composite. Initial KTAS was the arrival measure, ranging from level 1 to 5 [20,21,22]; reassessment frequency was not analysed. Medical presentation was a source indicator distinguishing disease-related visits from other recorded reasons, predominantly injury. Non-clinical visits and unspecified arrival/transport codes were enumerated and excluded in an additional sensitivity analysis.

Downstream outcomes were hospital admission, ICU admission, index-visit mortality and CPR. Index-visit mortality included recorded ED deaths, including dead-on-arrival encounters coded as deaths, and deaths during the linked admission. It did not capture deaths outside recorded encounters or after transfer or discharge. Daily downstream counts were assigned to the ED arrival date: ED-origin ICU admissions/day means visits on that date associated with subsequent ICU admission, not all ICU entries occurring on that calendar date or occupied ICU beds.

ED length of stay was arrival to actual ED departure. For admitted patients, admission-order issuance to ED departure was analysed as an order-to-departure interval, not a precise decision-to-transfer waiting time. Bed assignment was not treated as physical transfer. Official ICU records provided entry and exit dates in the available export, without clock times; their difference was reported in calendar days. Invalid sequences and unresolved intervals were excluded from interval summaries, with denominators shown in Table S13.

### 2.4. Comparative Models, Correlation and Multiplicity

A single model included crisis episode, phase and their interaction. The exponentiated interaction represented (workforce early/preceding)/(COVID-19 early/preceding). This observational ratio of relative changes was not interpreted as a causal difference-in-differences effect. Daily counts used Poisson regression with categorical calendar month and day of week; negative binomial regression assessed distributional robustness [23] (Table S3). Calendar-lag heteroscedasticity-and-autocorrelation-consistent variance used a 7-day Bartlett kernel, with 14-day sensitivity [24]. Calendar lags respected the actual gaps between historical windows.

Binary outcomes used modified Poisson regression [25,26]. The revised principal variance estimator clustered on both patient and actual ED arrival date, accounting for repeated visits and shared same-day conditions [27]. Patient-only, day-only and daily-score HAC(7)/HAC(14) estimates were also reported. Date clustering alone does not account for correlation between neighbouring dates; daily-score HAC addresses that distinct dependence assumption. None of these variance estimators supplies a concurrent unexposed comparator.

Nested comparisons comprised an unadjusted model, Model 0 (age, sex and calendar month), Model A (Model 0 plus the medical-presentation indicator), and Model B (Model A plus categorical initial KTAS and the five-component composite). Model A was used for front-door outcomes; Model B described downstream associations conditional on recorded initial acuity. Age was entered continuously in years. Downstream models were also compared on the identical complete-case population to distinguish adjustment from sample-selection changes. Models using the largest available sample were provided separately.

Medical presentation and initial acuity may themselves reflect crisis-related selection. Their inclusion standardises observed case mix but cannot identify a total causal effect [28]. Consequently, Model 0 and Model A are described as less-conditioned comparisons, not estimates of a total effect; Model B is explicitly acuity-conditioned. No single confirmatory primary endpoint had been designated among the original utilisation domains. These multi-outcome comparisons are exploratory. In response to review, Benjamini–Hochberg correction was added across the ten main interaction tests, while the nine diagnostic interactions formed a separate family [29]. Confidence intervals remain pointwise 95% intervals.

### 2.5. Longitudinal, Diagnostic and Sensitivity Analyses

Supplementary segmented Poisson analyses used the complete 2830-day series, with a linear time term and level and slope changes at 23 February 2020, 1 June 2023 and 20 February 2024. Categorical calendar month and day of week were included; two annual Fourier harmonics provided an alternative seasonal specification. Daily-score HAC(28) variance, residual diagnostics and descriptive comparisons of the 365-day pre-onset trends were reported. Separate daily-count and visit-offset models covered attendance, ED-origin ICU admissions, transfer-in proportion and admission proportion. These supplementary historical models do not remove unmeasured secular change or validate parallel trends.

Primary ED diagnoses at disposition defined nine fixed KCD/ICD-10 groups, analysed with daily visits as an offset (Table S5). Diagnosis rates describe composition per 1000 visits, not regional disease incidence. COVID-19 codes were reported separately because the pre-COVID-19 window had no U07-coded events. Categorical-month and Fourier models used the same diagnosis definitions and calendar-lag HAC(7) variance.

All ten main outcomes were re-estimated over 90-, 180- and 224-day windows with the same specification and confidence intervals. Additional reviewer-requested analyses used corresponding early-phase calendar dates in the previous year as baselines, excluded the 14 days before each onset, varied physiological completeness/definition, and compared correlation structures. The previous-year sensitivity used daily denominators, accommodating leap-year differences. All analyses used corrected source ages; uncertainty was recalculated rather than copied from the submission. Python 3.13.5, NumPy 2.3.5, pandas 2.2.3, SciPy 1.17.0 and statsmodels 0.14.6 were used.

## 3. Results

### 3.1. Population and Audit Findings

**Figure 1:**
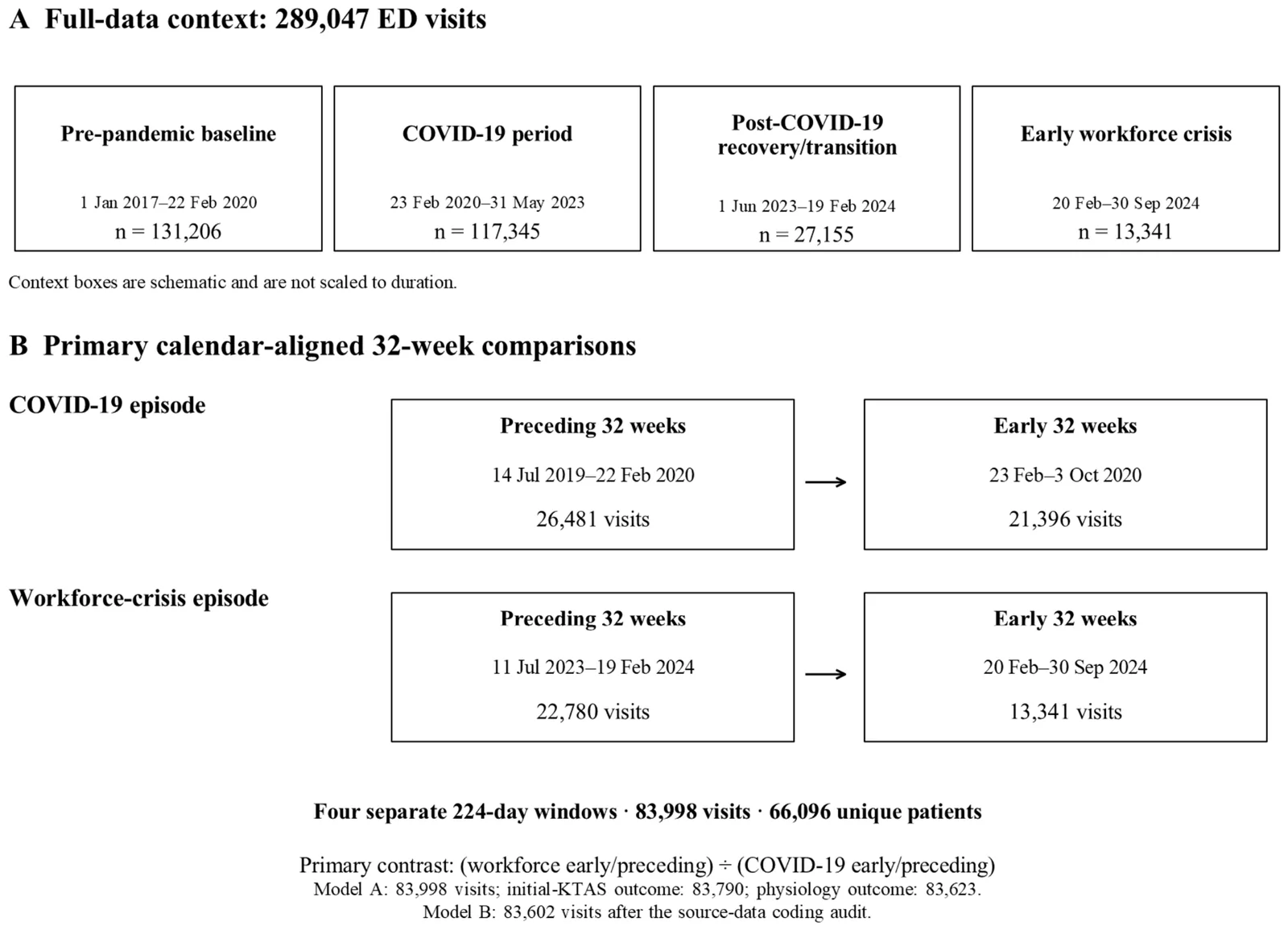
Study population and calendar-aligned analytical windows. Context boxes are schematic and not scaled to duration. The four separate 224-day windows total 896 analytical days. Patients may contribute to more than one window. Model denominators reflect corrected source-age parsing and the five-component recorded-physiology definition.

The full series included 289,047 registered ED visits. The primary windows contained 83,998 visits by 66,096 unique patients (Figure 1; Table 1). Recovery of month-coded infant ages increased the Model A population from 82,779 to 83,998. Initial KTAS was available for 83,790 visits, and at least one valid physiological component was available for 83,623. Model B included 83,602 visits after excluding unavailable KTAS or all-unavailable physiology; Table S4 separates overlapping exclusions and recording completeness.

Median ages across the windows were 53, 57, 59 and 63 years after age-unit correction. The five-component recorded-abnormality proportions were 23.5%, 21.6%, 25.5% and 25.3%, respectively. All five components were available in 79.5%, 87.2%, 88.2% and 90.8%. Component-specific abnormalities and completeness differed across eras (Table S12). The baseline populations were therefore not clinically or historically equivalent.

### 3.2. Observed and Adjusted Utilisation

**Figure 2:**
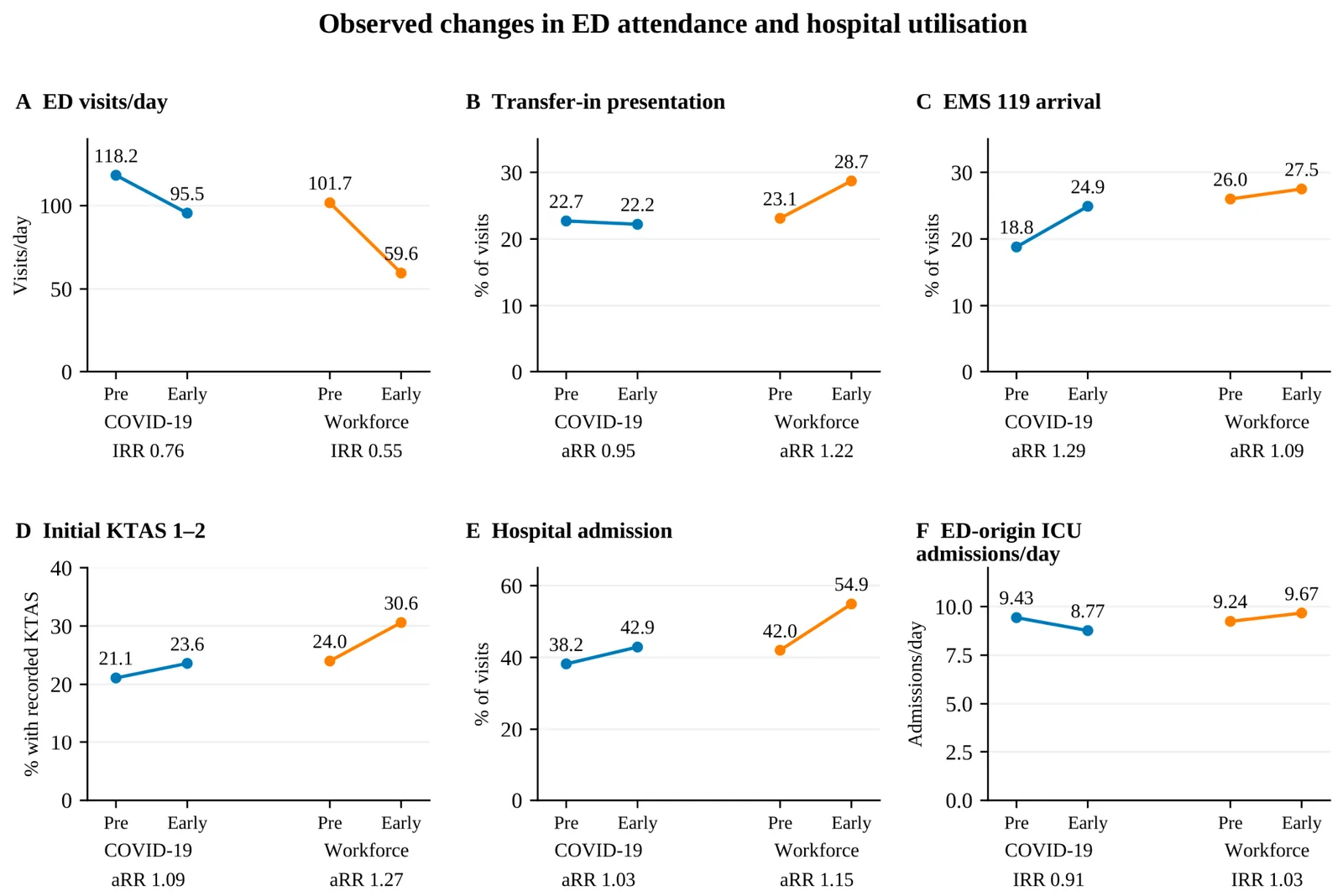
Unadjusted changes in attendance and hospital utilisation during the preceding and early windows. The two observations within each episode are connected for visual comparison; they are not a continuous longitudinal trajectory. Labels below panels give adjusted within-episode estimates. All y-axes start at zero and use panel-specific ranges. ED-origin ICU admissions/day counts subsequent ICU use among ED arrivals assigned to ED arrival date, not same-day ICU occupancy or all ICU entries. Pre, preceding window; ED, emergency department; EMS, emergency medical services; KTAS, Korean Triage and Acuity Scale; ICU, intensive care unit.

Daily attendance decreased from 118.2 to 95.5 during COVID-19 and from 101.7 to 59.6 during the workforce episode. Transfer-in proportion changed from 22.7% to 22.2% and from 23.1% to 28.7%, respectively. ICU admission proportions rose from 8.0% to 9.2% and from 9.1% to 16.2%; ED-origin ICU admissions/day changed from 9.43 to 8.77 and from 9.24 to 9.67 (Figure 2).

**Figure 3:**
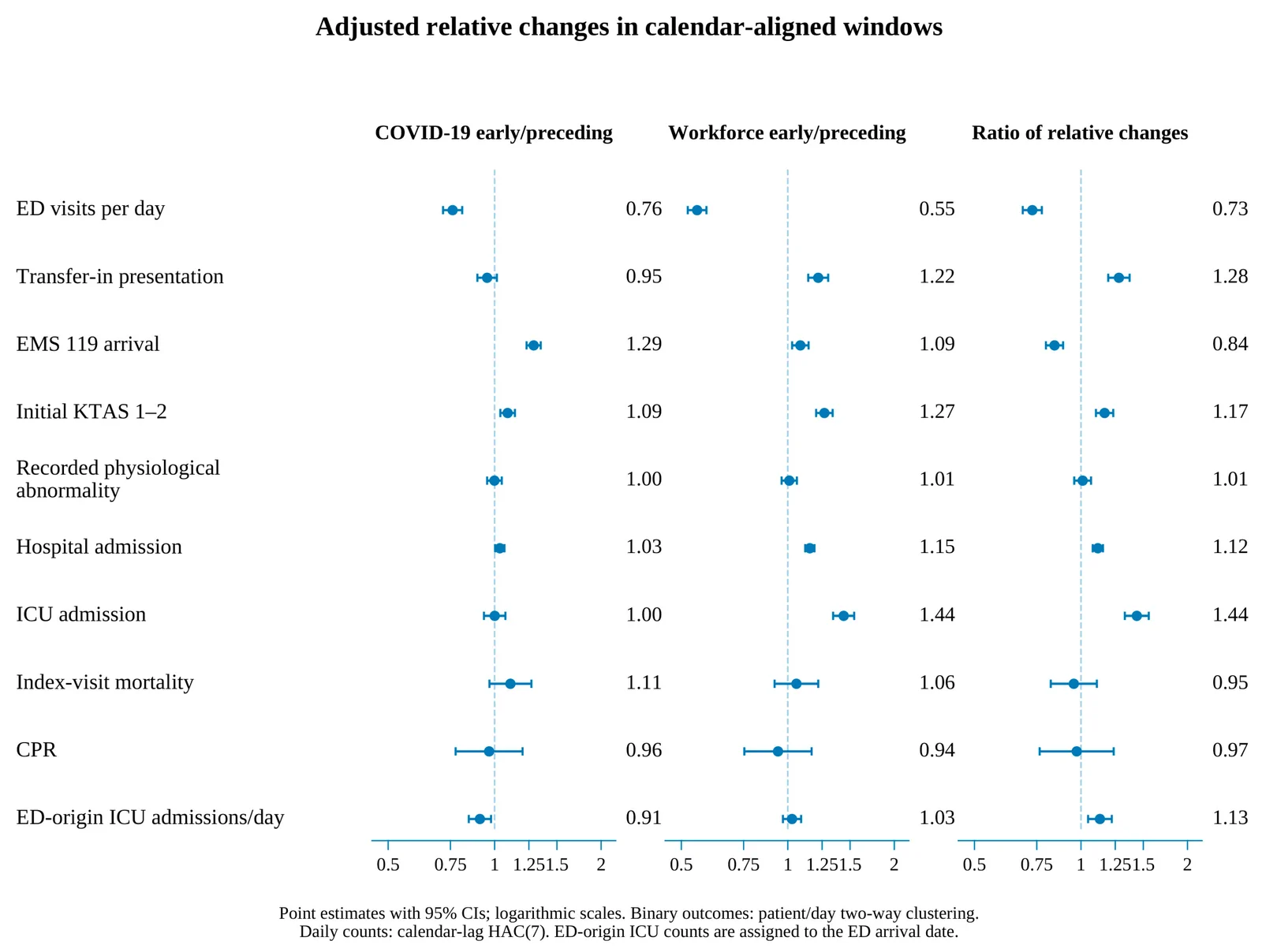
Revised main comparisons. All panels use logarithmic scales and identical row alignment. Error bars show pointwise 95% confidence intervals. Model A applies to transfer-in, EMS, KTAS and recorded physiology; Model B applies to hospital admission, ICU admission, mortality and CPR. Daily counts use calendar-lag HAC(7), and binary outcomes use patient-and-day two-way clustered variance. Numerical labels are point estimates; full intervals and multiplicity-adjusted interaction q values are presented in Table 2.

The adjusted attendance IRRs were 0.76 (95% CI, 0.71–0.81) and 0.55 (0.52–0.59), giving a relative-change ratio of 0.73 (0.68–0.78). Transfer-in aRRs were 0.95 (0.89–1.01) and 1.22 (1.14–1.30), respectively, with a ratio of 1.28 (1.19–1.37). Thus, the small COVID-19-period decrease no longer had a confidence interval excluding one under the revised analysis. EMS arrival increased more during COVID-19 (ratio, 0.84; 0.80–0.89). The KTAS 1–2 contrast was 1.17 (1.10–1.23), whereas the corrected physiological-composite contrast was 1.01 (0.96–1.07) (Table 2; Figure 3).

Under Model B, the between-episode ratios were 1.12 (1.08–1.15) for hospital admission and 1.44 (1.33–1.55) for ICU admission. Daily ED-origin ICU-count IRRs were 0.91 (0.85–0.98) during COVID-19 and 1.03 (0.97–1.09) during the workforce episode; their ratio was 1.13 (1.05–1.22). This was a comparison of observed utilisation, not an equivalence test or measurement of pressure. Absolute daily counts for all domains are presented in Table S2. The main interaction results for attendance, transfer-in, EMS, KTAS, admission, ICU admission and ED-origin ICU counts retained q < 0.05; physiology, mortality and CPR did not.

### 3.3. Adjustment, Mortality and Transfer-Out

On the common 83,602-visit sample, workforce-period mortality aRRs were 1.29 (1.11–1.50) in Model 0, 1.28 (1.10–1.49) in Model A and 1.06 (0.92–1.22) in Model B. The corresponding ICU admission aRRs were 1.68 (1.55–1.81), 1.70 (1.58–1.84) and 1.44 (1.34–1.54). The mortality finding therefore depended materially on conditioning on recorded initial acuity; it did not demonstrate unchanged survival (Table S8).

Overall ED transfer-out increased from 336/22,780 (1.47%) to 269/13,341 (2.02%), and mild-condition transfers increased from 34 (0.15%) to 59 (0.44%). Urgent/specialist/capacity-related transfers were 68 (0.30%) and 33 (0.25%). Combining only the latter transfers with index-visit death gave a Model B workforce aRR of 1.00 (0.87–1.15) (Table S7). This composite did not classify mild transfers as deaths and did not recover post-transfer outcomes. Excluding admissions with unresolved discharge dates gave a mortality aRR of 1.06 (0.92–1.22) but could not address unrecorded out-of-hospital deaths.

### 3.4. Temporal and Recording Sensitivities

Primary mean-model estimates were unchanged by the alternative variance estimators, while interval widths varied (Table S9). Transfer-in and acuity-conditioned ICU interaction directions were consistent across window lengths (Table S1; Figure S3). The physiological contrast was sensitive to definition and window: its 90-day ratio was 1.11 (1.02–1.20), but its 224-day ratio included one. Complete-five-component, strict-negative and four-component analyses were therefore retained without claiming universal physiological robustness (Table S12).

The full-period segmented analysis estimated an immediate workforce-onset attendance ratio of 0.72 (0.67–0.78) and an ED-origin ICU-count ratio of 1.13 (1.01–1.27). The subsequent attendance slope change was 0.97 per month (0.95–0.99). These level/slope estimates address a different question from the mean 224-day contrasts. Pre-onset monthly attendance slopes differed: 1.004 during the COVID-19-era baseline versus 0.976 during the workforce-era baseline, with a slope ratio of 0.973 (0.965–0.981) (Table S10; Figure S1). Parallel historical trends were not established.

Using corresponding early-phase dates in the previous year preserved the direction of the transfer-in and acuity-conditioned ICU contrasts (1.30 and 1.42, respectively), but the ED-origin ICU-count ratio was 1.06 (0.97–1.15). Therefore, the statistical distinction between episodes in absolute ICU flow was sensitive to baseline choice. Excluding the 14 pre-onset days gave an ICU-count ratio of 1.12 (1.04–1.22) (Table S11).

For descriptive process measures, workforce-period ED LOS shortened from a median 3.92 to 3.00 h, and the admission-order-to-departure interval among eligible admissions shortened from 1.60 to 1.00 h. The recorded ICU interval decreased from a median of 4 to 3 calendar days among valid, completed records. Coverage and timestamp limitations are reported in Table S13; these summaries do not demonstrate improved efficiency or quantify occupied-bed workload.

### 3.5. Diagnostic Composition

Changes in visit-normalised diagnostic composition were selective (Table 3; Figure S2). Pneumonia/aspiration decreased during early COVID-19, while respiratory failure/ARDS had a similar point-estimate direction but a confidence interval including one; both increased during the workforce episode. Gastrointestinal haemorrhage, including bleeding oesophageal varices, increased per 1000 visits in both episodes, with an aRR of 1.37 (1.18–1.60). Poisoning increased clearly only during the workforce episode, whereas traumatic intracranial injury showed opposite directions. The sepsis, myocardial-infarction and ischaemic-stroke interactions did not pass the nine-group FDR threshold. Fourier seasonal adjustment gave similar diagnostic interaction interpretations (Table S6).

## 4. Discussion

This single-centre comparison found a larger decline in attendance during the early workforce episode, accompanied by greater transfer-in and ICU admission proportions. ED-origin ICU admission volume did not fall detectably within that episode, although the contrast with COVID-19 depended partly on baseline choice. These observations support separating attendance, case mix and subsequent hospital utilisation; they do not establish pressure, capacity sufficiency or universal differences between crisis types.

The source audit materially changed one interpretation. Temperature had been omitted from the archived composite despite being specified in the submitted Methods, and infant month-coded ages had been lost in conversion. After correction, the five-component physiological contrast no longer distinguished the two 224-day transitions. Recording completeness and the individual components also varied across eras. The revised terminology, recorded physiological abnormalities, is deliberately narrower than instability or validated severity. The original coding is documented for reproducibility rather than retained to preserve a preferred result.

Calendar alignment addresses similar seasons, not historical exchangeability. The later baseline was older and differed in arrival and care patterns, and the extended analysis demonstrated differing pre-onset trends. Post-pandemic utilisation, hospital practices, referral roles and contemporaneous policies could contribute to the observed changes. The full-period segmented analysis strengthens temporal description but cannot isolate workforce withdrawal from these concurrent influences. Our inference is therefore restricted to the observed historical episodes.

ICU admissions are utilisation events. Their number does not specify staffing hours, occupancy, treatment intensity, admission thresholds or whether capacity was adequate. Moreover, our daily ICU measure is assigned to ED arrival date, rather than actual calendar-day ICU entry. Shorter recorded ED and ICU intervals further illustrate why admission counts alone cannot be translated into workload or pressure. Informal pre-registration redirection and changes in overall/mild-condition transfer-out could also alter the registered denominator. The low frequency of explicitly capacity-related transfers does not prove that capacity was adequate.

Adjustment deserves similar caution. Medical presentation and initial acuity may lie within crisis-related selection pathways; moving from Models 0/A to Model B changes the question. Less-conditioned workforce mortality estimates were higher, whereas the acuity-conditioned interval included one. The national study cited in the original submission reported a small increase in 30-day mortality after hospitalisation [8]. Its population, follow-up, comparison structure and outcome ascertainment differ from ours. Our index-encounter findings neither contradict that result nor exclude harm outside the centre or after discharge.

International application concerns the analytical approach, not the effect sizes. Other hospitals may have different referral roles, admission thresholds and responses to disrupted services. Monitoring attendance together with arrival route, documented acuity, admission proportions and ED-origin critical-care utilisation can describe changes missed by attendance alone. This study did not validate an early-warning tool or demonstrate how the same numerical changes would apply elsewhere.

### Limitations

The study lacked a concurrent unexposed hospital and included only two historical crisis episodes. Patient/day clustering and temporal variance estimators improve uncertainty estimation under specified dependence structures but cannot compensate for unmeasured secular differences or the small number of crisis episodes. Alternative baselines and segmented specifications did not eliminate these limitations.

Actual clinically available staffing and daily operational capacity could not be reliably reconstructed. Administrative employment records and nominal service-availability reports were not treated as clinical-capacity measurements. Informal screening/redirection was reported by investigators but lacked a dated institutional protocol, and its extent was not measured. National guidance, transfer incentives and later cost-sharing changes coincided with the workforce episode. The exposure consequently represents a period containing multiple changes rather than workforce reduction alone.

Routine recording differed across eras. Source validation identified and corrected age and composite-construction errors, but did not independently verify historical measurement practice. Partial physiology recording remains an imperfect basis for case-mix conditioning. Arrival and transport classifications included small, unspecified groups; complete-source sensitivities were consistent with the principal referral and admission findings. Diagnostic groups describe recorded composition, not individual disease severity or population incidence.

Index-visit mortality was limited to recorded ED/linked inpatient outcomes. Administrative transfer reasons cannot exclude clinically important deterioration after transfer. ICU interval analysis had incomplete coverage and calendar-date precision, and admission-order timing was only a proxy for a clinical decision. Repeated visits were retained and statistically clustered rather than removed. Finally, the workforce follow-up ended in September 2024 and does not describe the full disruption or later recovery.

## 5. Conclusions

During two calendar-aligned historical comparisons at one Korean regional emergency centre, ED attendance and subsequent hospital utilisation changed by different magnitudes. The workforce episode combined fewer visits with greater transfer-in and ICU admission proportions and no detectable within-episode decline in ED-origin ICU admission volume. The corrected physiological analysis, adjustment-dependent mortality findings and baseline-sensitive absolute-flow contrast constrain broader interpretation. The study supports multidimensional utilisation surveillance, not claims of quantified operational pressure, unchanged survival or universal crisis-specific responses.

## Figures and Tables

**Table 1:** Characteristics of the four analytical windows.

Characteristic	COVID-19 Preceding	COVID-19 Early	Workforce Preceding	Workforce Early
Study days	224	224	224	224
ED visits	26,481	21,396	22,780	13,341
Unique patients	22,640	18,516	19,700	11,497
ED visits/day	118.2	95.5	101.7	59.6
Age, years: median [IQR]	53 [22–70]	57 [32–73]	59 [31–74]	63 [42–76]
Male sex, *n* (%)	14,790 (55.9)	11,865 (55.5)	12,744 (55.9)	7452 (55.9)
Medical presentation	19,984 (75.5)	15,372 (71.8)	17,107 (75.1)	10,567 (79.2)
Transfer-in	6017 (22.7)	4745 (22.2)	5257 (23.1)	3828 (28.7)
EMS	4975 (18.8)	5336 (24.9)	5920 (26.0)	3675 (27.5)
Initial KTAS 1–2	5576/26,435 (21.1)	5039/21,352 (23.6)	5468/22,737 (24.0)	4063/13,266 (30.6)
Recorded physiology	6193/26,403 (23.5)	4605/21,336 (21.6)	5787/22,673 (25.5)	3338/13,211 (25.3)
Admission	10,128 (38.2)	9184 (42.9)	9561 (42.0)	7327 (54.9)
ICU admission	2113 (8.0)	1964 (9.2)	2070 (9.1)	2166 (16.2)
Mortality	638 (2.4)	628 (2.9)	739 (3.2)	551 (4.1)
CPR	249 (0.9)	229 (1.1)	319 (1.4)	233 (1.7)

Dates: COVID-19 Preceding, 14 July 2019–22 February 2020; COVID-19 Early, 23 February–3 October 2020; Workforce Preceding, 11 July 2023–19 February 2024; Workforce Early, 20 February–30 September 2024. Age includes source month-coded infant ages converted to years. Physiology uses the corrected five-component recorded-abnormality composite. KTAS and physiology percentages use the shown available denominators; other percentages use all visits. Per-window unique-patient counts overlap: 66,096 different patients contributed across all windows. IQR, interquartile range.

**Table 2:** Revised comparative estimates and interaction multiplicity adjustment.

Outcome	N *	COVID-19 Early/Preceding RR or IRR (95% CI)	Workforce Early/Preceding RR or IRR (95% CI)	Ratio of Relative Changes (95% CI)	Interaction q
ED visits/day	896	0.76 (0.71–0.81)	0.55 (0.52–0.59)	0.73 (0.68–0.78)	<0.001
Recorded transfer-in	83,998	0.95 (0.89–1.01)	1.22 (1.14–1.30)	1.28 (1.19–1.37)	<0.001
Recorded EMS 119 arrival	83,998	1.29 (1.23–1.35)	1.09 (1.03–1.14)	0.84 (0.80–0.89)	<0.001
Initial KTAS 1–2	83,790	1.09 (1.04–1.14)	1.27 (1.20–1.34)	1.17 (1.10–1.23)	<0.001
Recorded physiological abnormality	83,623	1.00 (0.95–1.05)	1.01 (0.96–1.06)	1.01 (0.96–1.07)	0.774
Hospital admission	83,602	1.03 (1.00–1.07)	1.15 (1.12–1.19)	1.12 (1.08–1.15)	<0.001
ICU admission	83,602	1.00 (0.93–1.07)	1.44 (1.34–1.54)	1.44 (1.33–1.55)	<0.001
Index-visit mortality	83,602	1.11 (0.97–1.27)	1.06 (0.92–1.22)	0.95 (0.82–1.11)	0.678
CPR	83,602	0.96 (0.78–1.20)	0.94 (0.75–1.17)	0.97 (0.76–1.24)	0.820
ED-origin ICU admissions/day	896	0.91 (0.85–0.98)	1.03 (0.97–1.09)	1.13 (1.05–1.22)	0.002

* N denotes visits for binary outcomes and analytical days for counts. Model A: age, sex, medical-presentation indicator and calendar month. Model B for admission, ICU admission, mortality and CPR adds initial KTAS and recorded physiological abnormalities. Binary confidence intervals use patient-and-day two-way clustered variance; count models use categorical calendar month, day of week and actual-calendar HAC(7). Interaction q values apply Benjamini–Hochberg correction to ten main comparisons and were added during revision; confidence intervals are pointwise, not simultaneous. ED-origin ICU counts are assigned to ED arrival date, not actual ICU entry date. Table S8 provides less-conditioned models.

**Table 3:** Nine-group diagnostic composition per 1000 ED visits.

Diagnosis	C pre n (Rate)	C Early n (Rate)	W pre n (Rate)	W Early n (Rate)	COVID-19 aRR (95% CI)	Workforce aRR (95% CI)	Ratio of aRRs (95% CI)	q
Acute upper respiratory infection	938 (35.4)	277 (12.9)	212 (9.3)	82 (6.1)	0.26 (0.19–0.35)	0.50 (0.36–0.70)	1.91 (1.40–2.62)	<0.001
Pneumonia/aspiration	773 (29.2)	211 (9.9)	435 (19.1)	350 (26.2)	0.47 (0.37–0.60)	1.85 (1.44–2.38)	3.94 (3.00–5.16)	<0.001
Respiratory failure/ARDS	73 (2.8)	34 (1.6)	99 (4.3)	129 (9.7)	0.65 (0.40–1.06)	2.48 (1.78–3.46)	3.82 (2.37–6.18)	<0.001
Sepsis/septic shock	171 (6.5)	129 (6.0)	201 (8.8)	141 (10.6)	0.99 (0.73–1.34)	1.27 (0.93–1.73)	1.28 (0.96–1.71)	0.119
Acute myocardial infarction	341 (12.9)	283 (13.2)	401 (17.6)	293 (22.0)	1.22 (0.96–1.56)	1.46 (1.21–1.76)	1.20 (0.95–1.50)	0.135
Ischaemic stroke	521 (19.7)	447 (20.9)	519 (22.8)	370 (27.7)	0.97 (0.81–1.16)	1.10 (0.92–1.32)	1.14 (0.95–1.36)	0.168
Gastrointestinal haemorrhage	574 (21.7)	511 (23.9)	591 (25.9)	521 (39.1)	1.23 (1.08–1.41)	1.69 (1.47–1.95)	1.37 (1.18–1.60)	<0.001
Poisoning/toxic effects	454 (17.1)	403 (18.8)	394 (17.3)	349 (26.2)	1.10 (0.92–1.32)	1.54 (1.30–1.82)	1.39 (1.15–1.69)	0.001
Traumatic intracranial injury	885 (33.4)	820 (38.3)	827 (36.3)	342 (25.6)	1.18 (1.04–1.33)	0.73 (0.64–0.83)	0.62 (0.53–0.72)	<0.001

Daily diagnosis counts used the log of daily ED visits as the offset, with episode, phase, interaction, categorical month, day of week and calendar-lag HAC(7) variance. Rates are composition measures per 1000 ED visits, not population incidence. The interaction q value is adjusted across all nine diagnostic groups. COVID-19 itself is not estimable against a zero-event pre-COVID-19 baseline. Residual seasonal and secular differences remain. aRR here denotes adjusted diagnosis rate ratio. The fixed codebook is presented in Table S5.

## Data Availability

Patient-level data are not publicly available because of institutional, ethical and privacy restrictions. Requests for access to de-identified data or analytic code may be directed to the corresponding author and considered subject to approval by the participating institution and its institutional review board.

## Supplementary Materials

The following supporting information can be downloaded at https://www.mdpi.com/article/10.3390/jcm15207783/s1: Methods S1; Tables S1–S14; Figures S1–S3. Supplementary methods include operational definitions, model equations, source-audit rules and all reviewer-requested sensitivity specifications.

## Abbreviations

The following abbreviations are used in this manuscript:

aRR	Adjusted risk ratio (binary outcomes) or adjusted rate ratio (diagnostic counts)
ARDS	Acute respiratory distress syndrome
CI	Confidence interval
COVID-19	Coronavirus disease 2019
CPR	Cardiopulmonary resuscitation
ED	Emergency department
EMS	Emergency medical services
FDR	False discovery rate
GCS	Glasgow Coma Scale
HAC	Heteroscedasticity-and-autocorrelation-consistent
ICD-10	International Classification of Diseases, 10th Revision
ICU	Intensive care unit
IQR	Interquartile range
IRR	Incidence rate ratio
KCD	Korean Standard Classification of Diseases
KTAS	Korean Triage and Acuity Scale
LOS	Length of stay
RECORD	REporting of studies Conducted using Observational Routinely-collected health Data
STROBE	Strengthening the Reporting of Observational Studies in Epidemiology
