# Supplementary material for: Emergency-Care Utilisation During Two Healthcare Crises in South Korea: A Single-Centre Comparison of Calendar-Aligned 32-Week Windows
Source: J Clin Med. 2026 Oct 9;15(20):7783. doi: 10.3390/jcm15207783 (PMC13705166; doi:10.3390/jcm15207783)
Supplement: Supplementary file 1 [file jcm-15-07783-s001.zip › jcm-4568328-supplementary.pdf]

Supplementary Material

Emergency-Care Utilisation During Two Healthcare Crises in South Korea: A Single-Centre Comparison of Calendar-Aligned 32-Week Windows

JCM-4568328 | Revised Methods S1, Tables S1–S14 and Figures S1–S3

Methods S1. Operational definitions, source audit and statistical model specification.

S1.1. Operational definitions and source coding

The source-data audit merged selected original ED, ICU/prescription and inpatient-discharge exports to the archived visit-level analytic file by the unique source encounter serial. Direct patient identifiers were retained only in the secure linkage environment and replaced with a study cluster identifier. Each ED source row and ICU-export row was unique by encounter serial. The full cohort contained 289,047 visits; the primary windows contained 83,998 visits by 66,096 patients. Source infant age strings 0m–11m were parsed as months/12, not treated as missing.

S1.2. Physiological measurements and partial missingness

Valid ranges were SBP >20 and ≤350 mmHg, heart rate >0 and ≤350/min, respiratory rate >0 and ≤100/min, temperature 25–45°C, and GCS 3–15. These are transparent data-cleaning screens, not a validated severity scale. Abnormality thresholds were applied consistently across ages rather than as age-specific paediatric triage criteria; an adult-only analysis assesses sensitivity to that choice. Negative sentinel values and zero values outside these rules were unavailable. Abnormality thresholds were SBP <90, HR >120, RR <10 or >30, temperature <35 or ≥38, or GCS ≤13. Any observed abnormality made the five-component proxy positive. At least one observed measurement with no abnormality made it 'no recorded abnormality'; this does not establish that unmeasured components were normal. All five unavailable made the proxy missing. A strict sensitivity kept positive cases with any abnormality but classified negatives only when all five were observed and normal. Other sensitivities used complete five-component records, the archived four-component proxy, adults only and adjustment for complete recording.

S1.3. Categorical variables and outcomes

Initial KTAS was the arrival level; codes outside 1–5 were unavailable. A blank reassessment meant no recorded change, but reassessment frequency was not an outcome. The medical-presentation indicator equalled one for disease-related visits and zero for other recorded reasons; the latter included 194 non-clinical primary-window encounters as well as injury. The original recorded transfer-in and EMS indicators were retained; 188 arrival-route source codes and 192 transport source values were unspecified. A sensitivity removed non-clinical encounters and records with unspecified route/transport. No source sex value was missing or outside M/F. In the primary windows, hospital admission exactly matched recorded ward transfer and ICU admission exactly matched a linked ICU flag or entry record. In the full-period data, 11 ICU flags had no archived hospital-admission flag and two admission flags differed from the ward-transfer code; none of these discordances occurred in the primary windows. Full-period supplementary models retain the recorded outcomes. Counts, denominator differences and exclusions are reported below.

S1.4. Time and mortality ascertainment

ED LOS was ED arrival to ED departure. Admission-order issuance to ED departure among admitted patients was an order-to-departure proxy. A valid interval required arrival ≤ order ≤ departure; negative or out-of-sequence intervals were not set to zero. Bed assignment did not mark physical movement and was not used as the endpoint. The available official ICU export had dates, not clock times, for ICU entry/exit. ICU intervals were exit date minus entry date, without adding one; same-date stays therefore had zero calendar-day difference. Valid completed ICU intervals required entry ≥ ED arrival date, exit ≥ entry and exit ≤ recorded hospital discharge date. Coverage is shown in Table

S13; these intervals are not total occupied-bed census. Index death was reconstructed from ED death/DOA dispositions and linked inpatient death; all four window death counts matched the submitted counts. Recorded DOA encounters were included, whereas out-of-hospital deaths without an ED record were not observed. An additional mortality sensitivity excluded unresolved admitted episodes and another excluded DOA.

S1.5. Mean models and estimands

For binary outcomes, $\log E(Y|X)=\beta 0+\beta W\ W+\beta P\ P+\beta WP(W\times P)+X\beta$. W denotes the workforce episode and P the early phase. $\exp(\beta P)$ is COVID early/preceding, $\exp(\beta P+\beta WP)$ is workforce early/preceding, and $\exp(\beta WP)$ is the ratio of relative changes. U includes only W, P and their interaction. Model 0 adds age in years (scaled by 10), sex and categorical month; A adds medical presentation; B adds categorical initial KTAS and the five-component proxy. Common-sample comparisons use exactly 83,602 visits. Available-sample comparisons use each model's maximum valid population. Daily models use the same episode/phase terms with month and day of week. Diagnosis counts use log(total daily ED visits) as an offset. The primary window models do not include a secular slope; full-series and historical-baseline sensitivities address that limitation separately.

S1.6. Variance and serial dependence

The principal binary variance is the inclusion–exclusion two-way cluster sandwich: patient-cluster variance plus date-cluster variance minus patient-by-date-intersection variance, with finite-cluster corrections. An independent comparison with statsmodels cov_cluster_2groups differed by less than 5×10^{-17} in the tested covariance matrix. Alternative variances are patient-only, date-only and HAC applied to daily sums of score contributions. Calendar-lag HAC uses Bartlett weights, respecting actual day distances; dates separated by the 2020–2023 gap are never treated as adjacent. Window count models use HAC(7); HAC(14) is supplementary. Full-series/pretrend models use HAC(28). Normal-approximation Wald intervals are pointwise. Corrected main GLMs converged with full-rank design matrices. Modified Poisson fitted means exceeded one in 519/83,602 admission records and 31/83,602 ICU records; these models estimate relative means and were not used as calibrated individual risk predictions.

S1.7. Longitudinal and baseline sensitivities

The complete daily series spans 1 January 2017–30 September 2024 (2,830 days); every day had a source ED record, so no unverified missing day was silently converted to zero. Segmented models include time in months (days/30.4375), indicators and time-since terms for COVID onset, the June 2023 transition and workforce onset, month and weekday. Fourier sensitivities replace month with first and second annual sine/cosine harmonics. Level estimates are discontinuities at the stated knots; slope-change estimates multiply the pre-existing monthly slope. They are not the same estimand as a 224-day average contrast. Monthly fitted means and delta-method confidence bands are aggregated from fitted daily means. Pretrend comparisons use the 365 days before each onset with separate linear episode slopes. The previous-calendar-year comparison replaces each preceding window with the same early-window calendar dates one year earlier; leap-year differences are retained and handled through daily denominators. Exclusion of the 14 days before each onset addresses possible anticipation but does not isolate all concurrent policies.

S1.8. Multiplicity, audit trail and analytical scope

The original study specified utilisation domains and a primary comparative interaction, but no single confirmatory primary outcome. Revision-stage analyses are exploratory. BH q values for ten main interactions were added in response to review; the nine diagnostic

interactions form a separate family. Nominal 95% intervals are not simultaneous intervals. The two episodes are not independent replications of crisis types. Administrative staffing counts, fluctuating nominal service restrictions and informal low-acuity redirection were not treated as quantified capacity measures. New enquiry/cancellation datasets were excluded. Source parsing, model functions, aggregate outputs and checksums are retained; no patient-level data are included in the revision package.

Table S1. Alternative window lengths with uncertainty estimates.

Days per window	Outcome	N*	COVID RR/IRR (95% CI)	Workforce RR/IRR (95% CI)	Ratio (95% CI)
90	ED visits/day	360	0.80 (0.72–0.90)	0.71 (0.63–0.81)	0.89 (0.82–0.97)
90	Recorded transfer-in	32237	0.87 (0.73–1.03)	1.09 (0.92–1.28)	1.25 (1.13–1.40)
90	Recorded EMS 119 arrival	32237	1.36 (1.16–1.59)	1.07 (0.92–1.25)	0.79 (0.72–0.87)
90	Initial KTAS 1–2	32154	1.01 (0.89–1.16)	1.08 (0.94–1.23)	1.06 (0.97–1.16)
90	Recorded physiological abnormality	32085	0.99 (0.89–1.11)	1.10 (0.98–1.24)	1.11 (1.02–1.20)
90	Hospital admission	32080	1.01 (0.93–1.09)	1.07 (1.00–1.16)	1.06 (1.01–1.12)
90	ICU admission	32080	1.05 (0.87–1.28)	1.52 (1.27–1.83)	1.45 (1.27–1.65)
90	Index-visit mortality	32080	0.99 (0.71–1.38)	0.93 (0.68–1.27)	0.94 (0.76–1.18)
90	CPR	32080	0.70 (0.39–1.27)	0.52 (0.30–0.93)	0.75 (0.52–1.08)
90	ED-origin ICU admissions/day	360	0.88 (0.76–1.01)	1.08 (0.95–1.22)	1.23 (1.08–1.40)
180	ED visits/day	720	0.82 (0.76–0.89)	0.62 (0.57–0.67)	0.75 (0.70–0.80)
180	Recorded transfer-in	67397	0.85 (0.76–0.96)	1.07 (0.96–1.20)	1.26 (1.16–1.36)
180	Recorded EMS 119 arrival	67397	1.31 (1.19–1.44)	1.09 (0.99–1.21)	0.84 (0.79–0.89)
180	Initial KTAS 1–2	67228	1.08 (0.99–1.18)	1.25 (1.14–1.37)	1.16 (1.09–1.23)
180	Recorded physiological abnormality	67096	1.03 (0.95–1.12)	1.08 (0.99–1.17)	1.05 (0.98–1.11)
180	Hospital admission	67080	0.99 (0.94–1.05)	1.08 (1.03–1.14)	1.09 (1.05–1.13)
180	ICU admission	67080	1.03 (0.90–1.17)	1.49 (1.32–1.67)	1.45 (1.33–1.58)
180	Index-visit mortality	67080	0.96 (0.74–1.25)	0.97 (0.75–1.25)	1.01 (0.86–1.20)
180	CPR	67080	1.02 (0.71–1.47)	0.96 (0.68–1.34)	0.94 (0.72–1.22)
180	ED-origin ICU admissions/day	720	0.96 (0.87–1.05)	1.09 (1.00–1.18)	1.14 (1.04–1.24)
224	ED visits/day	896	0.76 (0.71–0.81)	0.55 (0.52–0.59)	0.73 (0.68–0.78)
224	Recorded transfer-in	83998	0.95 (0.89–1.01)	1.22 (1.14–1.30)	1.28 (1.19–1.37)
224	Recorded EMS 119 arrival	83998	1.29 (1.23–1.35)	1.09 (1.03–1.14)	0.84 (0.80–0.89)
224	Initial KTAS 1–2	83790	1.09 (1.04–1.14)	1.27 (1.20–1.34)	1.17 (1.10–1.23)
224	Recorded physiological abnormality	83623	1.00 (0.95–1.05)	1.01 (0.96–1.06)	1.01 (0.96–1.07)
224	Hospital admission	83602	1.03 (1.00–1.07)	1.15 (1.12–1.19)	1.12 (1.08–1.15)
224	ICU admission	83602	1.00 (0.93–1.07)	1.44 (1.34–1.54)	1.44 (1.33–1.55)
224	Index-visit mortality	83602	1.11 (0.97–1.27)	1.06 (0.92–1.22)	0.95 (0.82–1.11)
224	CPR	83602	0.96 (0.78–1.20)	0.94 (0.75–1.17)	0.97 (0.76–1.24)
224	ED-origin ICU admissions/day	896	0.91 (0.85–0.98)	1.03 (0.97–1.09)	1.13 (1.05–1.22)

* Visits or days as appropriate. The 224-day rows exactly match Table 2. Same categorical-month specification and corrected source variables across all durations; no switching to Fourier adjustment between tables.

Table S2. Absolute ED-origin daily counts.

Outcome, events/day	COVID IRR (95% CI)	Workforce IRR (95% CI)	Ratio of IRRs (95% CI)
ED visits/day	0.76 (0.71–0.81)	0.55 (0.52–0.59)	0.73 (0.68–0.78)
Recorded transfer-in	0.76 (0.72–0.81)	0.71 (0.67–0.75)	0.93 (0.87–0.98)
Recorded EMS 119 arrival	1.06 (1.01–1.12)	0.62 (0.58–0.66)	0.58 (0.55–0.62)
Initial KTAS 1–2	0.89 (0.84–0.94)	0.73 (0.69–0.77)	0.83 (0.78–0.88)
Recorded physiological abnormality	0.70 (0.64–0.76)	0.54 (0.49–0.60)	0.78 (0.71–0.86)
Hospital admission	0.87 (0.83–0.91)	0.74 (0.71–0.76)	0.85 (0.81–0.89)
ICU admission	0.91 (0.85–0.98)	1.03 (0.97–1.09)	1.13 (1.05–1.22)
Index-visit mortality	1.05 (0.93–1.20)	0.80 (0.69–0.93)	0.76 (0.66–0.88)
CPR	0.94 (0.77–1.15)	0.75 (0.59–0.95)	0.79 (0.62–1.01)

All downstream events are attributed to the ED arrival date. These are not actual calendar-day ward/ICU census or total hospital ICU entries. The physiology count is the number with a recorded abnormality, not an imputed count for missing measurements.

Table S3. Daily-volume specification and distributional audit.

Specification	COVID IRR (95% CI)	Workforce IRR (95% CI)	Ratio (95% CI)
Poisson_month	0.76 (0.71–0.81)	0.55 (0.52–0.59)	0.73 (0.68–0.78)
Poisson_fourier	0.76 (0.71–0.80)	0.55 (0.52–0.58)	0.73 (0.68–0.78)
Negative_binomial_month	0.76 (0.71–0.81)	0.55 (0.52–0.59)	0.73 (0.69–0.78)

Categorical-month Poisson point estimates before rounding: 0.760652 and 0.554083. Fourier Poisson: 0.757561 and 0.551046. This exactly explains the submitted Table S1/S3 point-estimate discrepancy; it was not rounding. The revised duration analysis uses categorical month throughout. Negative-binomial alpha ≈0.00852. All rows now use actual-calendar HAC(7).

Table S4. Missingness, recoverable source ages and model denominators.

Variable: unavailable n/N (%)	COVID preceding	COVID early	Workforce preceding	Workforce early
Age	0/26,481 (0.00%)	0/21,396 (0.00%)	0/22,780 (0.00%)	0/13,341 (0.00%)
Initial KTAS	46/26,481 (0.17%)	44/21,396 (0.21%)	43/22,780 (0.19%)	75/13,341 (0.56%)
SBP	5,118/26,481 (19.33%)	2,497/21,396 (11.67%)	2,399/22,780 (10.53%)	1,051/13,341 (7.88%)
Heart rate	4,840/26,481 (18.28%)	2,382/21,396 (11.13%)	375/22,780 (1.65%)	291/13,341 (2.18%)
Respiratory rate	5,020/26,481 (18.96%)	2,490/21,396 (11.64%)	1,019/22,780 (4.47%)	366/13,341 (2.74%)
Temperature	115/26,481 (0.43%)	130/21,396 (0.61%)	109/22,780 (0.48%)	134/13,341 (1.00%)
GCS	395/26,481 (1.49%)	300/21,396 (1.40%)	486/22,780 (2.13%)	379/13,341 (2.84%)
Five-component proxy	78/26,481 (0.29%)	60/21,396 (0.28%)	107/22,780 (0.47%)	130/13,341 (0.97%)
Sex	0/26,481 (0.00%)	0/21,396 (0.00%)	0/22,780 (0.00%)	0/13,341 (0.00%)
Medical presentation indicator	0/26,481 (0.00%)	0/21,396 (0.00%)	0/22,780 (0.00%)	0/13,341 (0.00%)
Arrival route source	45/26,481 (0.17%)	38/21,396 (0.18%)	35/22,780 (0.15%)	70/13,341 (0.52%)
Transport source	45/26,481 (0.17%)	39/21,396 (0.18%)	35/22,780 (0.15%)	73/13,341 (0.55%)
Audit item	COVID preceding	COVID early	Workforce preceding	Workforce early
Window visits	26,481	21,396	22,780	13,341
Recovered month-coded ages	549	245	295	130
Original Model A	25,932	21,151	22,485	13,211
Revised Model A	26,481	21,396	22,780	13,341
Original Model B	25,927	21,145	22,475	13,210
Revised Model B	26,398	21,331	22,663	13,210
KTAS unavailable	46	44	43	75
All five physiological measurements unavailable	78	60	107	130
All five measured	21,062	18,661	20,094	12,114
Incomplete with no recorded abnormality	3,149	1,825	806	340

The 1,219 originally excluded age values were valid month-coded infant ages, not imputed unknowns. After correction, no primary-window age or sex was missing. Revised Model B excludes 396 distinct visits: 208 with unavailable KTAS and 375 with all physiology unavailable, with overlap. Unknown route/transport values are enumerated separately and excluded in Table S12 sensitivity, not concealed as missing covariates.

Table S5. Fixed diagnostic codebook.

Group	KCD/ICD-10 definition
Acute upper respiratory infection	J00–J06
Pneumonia/aspiration	J12–J18; J69
Respiratory failure/ARDS	J80; J96
Sepsis/septic shock	A40–A41; R57.2; R65.2
Acute myocardial infarction	I21–I22
Ischaemic stroke	I63 (TIA and unspecified stroke excluded)
Gastrointestinal haemorrhage	I85.0; K22.6; haemorrhagic K25–K28 subcodes with fourth digit 0, 2, 4 or 6; K29.0; K62.5; K92.0–K92.2
Poisoning/toxic effects	T36–T65; intent codes were not used
Traumatic intracranial injury	S06
COVID-19, separately described	U07.1–U07.2; no estimable pre-COVID ratio

Principal ED disposition diagnosis only. Codes are uppercased, punctuation removed and prefixes matched. Bleeding oesophageal varices are included; nonbleeding varices are excluded. R65.2 belongs to the fixed code list but no such primary-window event was present. Diagnostic definitions did not change in this revision.

Table S6. Diagnostic seasonality sensitivity.

Diagnosis	Seasonality	COVID aRR (95% CI)	Workforce aRR (95% CI)	Ratio (95% CI)	Interaction q
Acute upper respiratory infection	month	0.26 (0.19–0.35)	0.50 (0.36–0.70)	1.91 (1.40–2.62)	<0.001
Acute upper respiratory infection	fourier	0.26 (0.20–0.35)	0.50 (0.36–0.70)	1.91 (1.39–2.63)	<0.001
Pneumonia/aspiration	month	0.47 (0.37–0.60)	1.85 (1.44–2.38)	3.94 (3.00–5.16)	<0.001
Pneumonia/aspiration	fourier	0.49 (0.39–0.63)	1.95 (1.53–2.48)	3.96 (3.00–5.23)	<0.001
Respiratory failure/ARDS	month	0.65 (0.40–1.06)	2.48 (1.78–3.46)	3.82 (2.37–6.18)	<0.001
Respiratory failure/ARDS	fourier	0.60 (0.37–0.98)	2.29 (1.63–3.21)	3.82 (2.34–6.25)	<0.001
Sepsis/septic shock	month	0.99 (0.73–1.34)	1.27 (0.93–1.73)	1.28 (0.96–1.71)	0.119
Sepsis/septic shock	fourier	1.00 (0.73–1.36)	1.27 (0.94–1.70)	1.27 (0.94–1.72)	0.143
Acute myocardial infarction	month	1.22 (0.96–1.56)	1.46 (1.21–1.76)	1.20 (0.95–1.50)	0.135
Acute myocardial infarction	fourier	1.20 (0.95–1.53)	1.44 (1.19–1.73)	1.19 (0.95–1.50)	0.143
Ischaemic stroke	month	0.97 (0.81–1.16)	1.10 (0.92–1.32)	1.14 (0.95–1.36)	0.168
Ischaemic stroke	fourier	0.99 (0.83–1.19)	1.12 (0.94–1.34)	1.13 (0.94–1.36)	0.180
Gastrointestinal haemorrhage	month	1.23 (1.08–1.41)	1.69 (1.47–1.95)	1.37 (1.18–1.60)	<0.001
Gastrointestinal haemorrhage	fourier	1.22 (1.06–1.41)	1.68 (1.43–1.97)	1.37 (1.16–1.61)	<0.001
Poisoning/toxic effects	month	1.10 (0.92–1.32)	1.54 (1.30–1.82)	1.39 (1.15–1.69)	0.001
Poisoning/toxic effects	fourier	1.12 (0.92–1.37)	1.56 (1.29–1.89)	1.39 (1.12–1.73)	0.004
Traumatic intracranial injury	month	1.18 (1.04–1.33)	0.73 (0.64–0.83)	0.62 (0.53–0.72)	<0.001
Traumatic intracranial injury	fourier	1.19 (1.06–1.34)	0.74 (0.64–0.84)	0.62 (0.53–0.72)	<0.001

Table S7. Transfer-out reasons and limited mortality sensitivity.

Reason, n (% of all ED visits)	COVID preceding	COVID early	Workforce preceding	Workforce early
Patient/family circumstances	266 (1.00%)	156 (0.73%)	161 (0.71%)	104 (0.78%)
Return transfer	32 (0.12%)	17 (0.08%)	34 (0.15%)	26 (0.19%)
Mild-condition transfer	29 (0.11%)	7 (0.03%)	34 (0.15%)	59 (0.44%)
Long-term-care transfer	11 (0.04%)	5 (0.02%)	26 (0.11%)	21 (0.16%)
Emergency treatment unavailable	31 (0.12%)	15 (0.07%)	37 (0.16%)	17 (0.13%)
Specialist emergency care required	23 (0.09%)	27 (0.13%)	21 (0.09%)	16 (0.12%)
Ward bed unavailable	5 (0.02%)	0 (0.00%)	8 (0.04%)	0 (0.00%)
ICU bed unavailable	4 (0.02%)	3 (0.01%)	2 (0.01%)	0 (0.00%)
Other/unspecified transfer	2 (0.01%)	0 (0.00%)	13 (0.06%)	26 (0.19%)
Any ED transfer-out	403 (1.52%)	230 (1.07%)	336 (1.47%)	269 (2.02%)

Urgent/specialist/capacity-related = immediate emergency surgery/treatment unavailable, specialist emergency care required, ward unavailable or ICU unavailable. Explicit capacity excludes specialist-care-only requests. Mild/long-term-care/patient-request transfers were not classified as deaths.

Model B outcome	COVID aRR (95% CI)	Workforce aRR (95% CI)	Ratio (95% CI)
Any ED transfer-out	0.59 (0.48–0.74)	1.14 (0.93–1.41)	1.93 (1.53–2.42)
Urgent/specialist/capacity transfer-out	0.57 (0.34–0.95)	0.51 (0.28–0.91)	0.89 (0.50–1.58)
Explicit capacity-related transfer-out	0.37 (0.19–0.74)	0.40 (0.18–0.88)	1.08 (0.49–2.37)
Death or urgent/specialist/capacity transfer-out	1.05 (0.93–1.20)	1.00 (0.87–1.15)	0.95 (0.82–1.10)
Death or explicit capacity-related transfer-out	1.05 (0.92–1.20)	1.00 (0.87–1.15)	0.96 (0.83–1.11)

These post-arrival disposition sensitivities use the revised age/composite and two-way variance. Administrative reason codes do not establish clinical stability after transfer. The composite is a separate event definition, not recovered mortality.

Table S8. Sequential adjustment on common and available samples.

Population	Outcome	Model	N	COVID RR (95% CI)	Workforce RR (95% CI)	Ratio (95% CI)
common	Hospital admission	U	83602	1.12 (1.08–1.16)	1.31 (1.26–1.35)	1.16 (1.11–1.22)
common	Hospital admission	M0	83602	1.07 (1.03–1.11)	1.25 (1.20–1.29)	1.17 (1.13–1.22)
common	Hospital admission	A	83602	1.08 (1.04–1.12)	1.23 (1.19–1.27)	1.14 (1.10–1.19)
common	Hospital admission	B	83602	1.03 (1.00–1.07)	1.15 (1.12–1.19)	1.12 (1.08–1.15)
common	ICU admission	U	83602	1.15 (1.08–1.23)	1.76 (1.65–1.88)	1.53 (1.39–1.68)
common	ICU admission	M0	83602	1.09 (1.01–1.17)	1.68 (1.55–1.81)	1.55 (1.42–1.68)
common	ICU admission	A	83602	1.08 (1.00–1.16)	1.70 (1.58–1.84)	1.58 (1.45–1.73)
common	ICU admission	B	83602	1.00 (0.93–1.07)	1.44 (1.34–1.54)	1.44 (1.33–1.55)
common	Index-visit mortality	U	83602	1.25 (1.11–1.39)	1.28 (1.14–1.44)	1.03 (0.87–1.21)
common	Index-visit mortality	M0	83602	1.24 (1.08–1.43)	1.29 (1.11–1.50)	1.04 (0.89–1.22)
common	Index-visit mortality	A	83602	1.24 (1.08–1.43)	1.28 (1.10–1.49)	1.03 (0.88–1.21)
common	Index-visit mortality	B	83602	1.11 (0.97–1.27)	1.06 (0.92–1.22)	0.95 (0.82–1.11)
common	CPR	U	83602	1.14 (0.95–1.37)	1.23 (1.03–1.47)	1.08 (0.84–1.40)
common	CPR	M0	83602	1.10 (0.88–1.37)	1.19 (0.95–1.50)	1.09 (0.84–1.40)
common	CPR	A	83602	1.10 (0.88–1.37)	1.19 (0.94–1.50)	1.08 (0.84–1.40)
common	CPR	B	83602	0.96 (0.78–1.20)	0.94 (0.75–1.17)	0.97 (0.76–1.24)
common	Recorded transfer-in	M0	83602	0.95 (0.89–1.01)	1.23 (1.15–1.31)	1.29 (1.20–1.38)
common	Recorded transfer-in	A	83602	0.95 (0.89–1.01)	1.22 (1.15–1.30)	1.28 (1.20–1.38)
common	Recorded EMS 119 arrival	M0	83602	1.32 (1.25–1.38)	1.06 (1.01–1.12)	0.81 (0.76–0.86)
common	Recorded EMS 119 arrival	A	83602	1.29 (1.23–1.35)	1.09 (1.03–1.15)	0.84 (0.80–0.89)
available	Hospital admission	U	83998	1.12 (1.08–1.16)	1.31 (1.27–1.35)	1.17 (1.11–1.22)
available	Hospital admission	M0	83998	1.07 (1.03–1.11)	1.26 (1.21–1.30)	1.18 (1.13–1.22)
available	Hospital admission	A	83998	1.08 (1.04–1.12)	1.24 (1.20–1.28)	1.15 (1.10–1.19)
available	Hospital admission	B	83602	1.03 (1.00–1.07)	1.15 (1.12–1.19)	1.12 (1.08–1.15)
available	ICU admission	U	83998	1.15 (1.08–1.23)	1.79 (1.67–1.91)	1.55 (1.42–1.70)
available	ICU admission	M0	83998	1.10 (1.02–1.18)	1.72 (1.60–1.86)	1.57 (1.44–1.71)
available	ICU admission	A	83998	1.08 (1.00–1.17)	1.76 (1.63–1.89)	1.62 (1.49–1.76)
available	ICU admission	B	83602	1.00 (0.93–1.07)	1.44 (1.34–1.54)	1.44 (1.33–1.55)
available	Index-visit mortality	U	83998	1.22 (1.09–1.36)	1.27 (1.14–1.42)	1.05 (0.90–1.22)
available	Index-visit mortality	M0	83998	1.21 (1.05–1.38)	1.28 (1.11–1.48)	1.06 (0.91–1.23)
available	Index-visit mortality	A	83998	1.21 (1.05–1.38)	1.28 (1.11–1.47)	1.06 (0.91–1.23)
available	Index-visit mortality	B	83602	1.11 (0.97–1.27)	1.06 (0.92–1.22)	0.95 (0.82–1.11)
available	CPR	U	83998	1.14 (0.95–1.37)	1.25 (1.04–1.49)	1.10 (0.85–1.41)

Population	Outcome	Model	N	COVID RR (95% CI)	Workforce RR (95% CI)	Ratio (95% CI)
available	CPR	M0	83998	1.10 (0.88–1.38)	1.22 (0.97–1.53)	1.10 (0.86–1.42)
available	CPR	A	83998	1.10 (0.88–1.38)	1.22 (0.97–1.53)	1.10 (0.86–1.42)
available	CPR	B	83602	0.96 (0.78–1.20)	0.94 (0.75–1.17)	0.97 (0.76–1.24)
available	Recorded transfer-in	M0	83998	0.95 (0.89–1.01)	1.22 (1.14–1.30)	1.29 (1.20–1.38)
available	Recorded transfer-in	A	83998	0.95 (0.89–1.01)	1.22 (1.14–1.30)	1.28 (1.19–1.37)
available	Recorded EMS 119 arrival	M0	83998	1.31 (1.25–1.38)	1.06 (1.00–1.12)	0.81 (0.76–0.85)
available	Recorded EMS 119 arrival	A	83998	1.29 (1.23–1.35)	1.09 (1.03–1.14)	0.84 (0.80–0.89)

U: episode, phase, interaction only. Model 0: age, sex, month. A: adds medical presentation. B: adds initial KTAS and recorded physiology. Common sample = 83,602 visits for all rows, preventing different samples from masquerading as adjustment effects. None of the models identifies a total causal effect.

Table S9. Correlation-structure sensitivity.

Outcome	Variance estimator	Ratio (95% CI)	Nominal P
ED visits/day	HAC7	0.73 (0.68–0.78)	<0.001
ED visits/day	HAC14	0.73 (0.68–0.78)	<0.001
Recorded transfer-in	patient	1.28 (1.22–1.35)	<0.001
Recorded transfer-in	day	1.28 (1.20–1.37)	<0.001
Recorded transfer-in	twoway	1.28 (1.19–1.37)	<0.001
Recorded transfer-in	HAC7	1.28 (1.21–1.35)	<0.001
Recorded transfer-in	HAC14	1.28 (1.21–1.35)	<0.001
Recorded EMS 119 arrival	patient	0.84 (0.80–0.89)	<0.001
Recorded EMS 119 arrival	day	0.84 (0.80–0.89)	<0.001
Recorded EMS 119 arrival	twoway	0.84 (0.80–0.89)	<0.001
Recorded EMS 119 arrival	HAC7	0.84 (0.79–0.89)	<0.001
Recorded EMS 119 arrival	HAC14	0.84 (0.79–0.89)	<0.001
Initial KTAS 1–2	patient	1.17 (1.11–1.22)	<0.001
Initial KTAS 1–2	day	1.17 (1.10–1.23)	<0.001
Initial KTAS 1–2	twoway	1.17 (1.10–1.23)	<0.001
Initial KTAS 1–2	HAC7	1.17 (1.10–1.24)	<0.001
Initial KTAS 1–2	HAC14	1.17 (1.10–1.24)	<0.001
Recorded physiological abnormality	patient	1.01 (0.96–1.06)	0.676
Recorded physiological abnormality	day	1.01 (0.96–1.07)	0.687
Recorded physiological abnormality	twoway	1.01 (0.96–1.07)	0.697
Recorded physiological abnormality	HAC7	1.01 (0.95–1.07)	0.728
Recorded physiological abnormality	HAC14	1.01 (0.95–1.08)	0.750
Hospital admission	patient	1.12 (1.08–1.15)	<0.001
Hospital admission	day	1.12 (1.08–1.15)	<0.001
Hospital admission	twoway	1.12 (1.08–1.15)	<0.001
Hospital admission	HAC7	1.12 (1.08–1.16)	<0.001
Hospital admission	HAC14	1.12 (1.08–1.16)	<0.001
ICU admission	patient	1.44 (1.33–1.55)	<0.001
ICU admission	day	1.44 (1.33–1.55)	<0.001
ICU admission	twoway	1.44 (1.33–1.55)	<0.001
ICU admission	HAC7	1.44 (1.33–1.55)	<0.001
ICU admission	HAC14	1.44 (1.33–1.56)	<0.001
Index-visit mortality	patient	0.95 (0.82–1.11)	0.538

Outcome	Variance estimator	Ratio (95% CI)	Nominal P
Index-visit mortality	day	0.95 (0.82–1.11)	0.543
Index-visit mortality	twoway	0.95 (0.82–1.11)	0.542
Index-visit mortality	HAC7	0.95 (0.82–1.11)	0.535
Index-visit mortality	HAC14	0.95 (0.83–1.10)	0.523
CPR	patient	0.97 (0.77–1.23)	0.818
CPR	day	0.97 (0.76–1.24)	0.820
CPR	twoway	0.97 (0.76–1.24)	0.820
CPR	HAC7	0.97 (0.77–1.23)	0.816
CPR	HAC14	0.97 (0.77–1.23)	0.817
ED-origin ICU admissions/day	HAC7	1.13 (1.05–1.22)	0.002
ED-origin ICU admissions/day	HAC14	1.13 (1.05–1.22)	<0.001

Same data and mean model for every variance estimator within an outcome. Two-way = patient plus day minus patient-by-day intersection. Daily-score HAC uses actual calendar lags; no artificial adjacency across gaps. These covariance alternatives do not solve historical confounding.

Table S10. Full-period segmented models and pre-onset trends.

Outcome	Seasonality	Knot	Parameter	Estimate (95% CI)	P
visits	month	covid	level	0.740 (0.693–0.790)	<0.001
visits	month	covid	slope change per month	1.007 (1.004–1.009)	<0.001
visits	month	recovery	level	0.954 (0.896–1.016)	0.146
visits	month	recovery	slope change per month	0.980 (0.969–0.992)	<0.001
visits	month	workforce	level	0.719 (0.666–0.777)	<0.001
visits	month	workforce	slope change per month	0.967 (0.948–0.985)	<0.001
icu	month	covid	level	0.877 (0.814–0.945)	<0.001
icu	month	covid	slope change per month	1.002 (0.998–1.005)	0.306
icu	month	recovery	level	1.081 (0.986–1.185)	0.098
icu	month	recovery	slope change per month	0.986 (0.972–1.000)	0.056
icu	month	workforce	level	1.135 (1.011–1.274)	0.033
icu	month	workforce	slope change per month	0.995 (0.970–1.022)	0.735
transfer	month	covid	level	0.974 (0.936–1.014)	0.207
transfer	month	covid	slope change per month	1.001 (0.999–1.003)	0.306
transfer	month	recovery	level	1.052 (0.971–1.139)	0.218
transfer	month	recovery	slope change per month	1.001 (0.989–1.014)	0.837
transfer	month	workforce	level	1.116 (1.046–1.190)	<0.001
transfer	month	workforce	slope change per month	1.023 (1.005–1.043)	0.015
admission	month	covid	level	1.151 (1.108–1.195)	<0.001
admission	month	covid	slope change per month	0.997 (0.996–0.999)	<0.001
admission	month	recovery	level	1.070 (1.016–1.127)	0.011
admission	month	recovery	slope change per month	0.997 (0.988–1.006)	0.555
admission	month	workforce	level	1.138 (1.077–1.202)	<0.001
admission	month	workforce	slope change per month	1.047 (1.034–1.061)	<0.001
visits	fourier	covid	level	0.737 (0.690–0.787)	<0.001
visits	fourier	covid	slope change per month	1.007 (1.004–1.009)	<0.001
visits	fourier	recovery	level	0.943 (0.884–1.006)	0.076
visits	fourier	recovery	slope change per month	0.982 (0.971–0.994)	0.003
visits	fourier	workforce	level	0.705 (0.656–0.757)	<0.001
visits	fourier	workforce	slope change per month	0.967 (0.949–0.985)	<0.001
icu	fourier	covid	level	0.878 (0.814–0.947)	<0.001
icu	fourier	covid	slope change per month	1.002 (0.998–1.005)	0.325
icu	fourier	recovery	level	1.080 (0.984–1.184)	0.105

Outcome	Seasonality	Knot	Parameter	Estimate (95% CI)	P
icu	fourier	recovery	slope change per month	0.986 (0.971–1.002)	0.077
icu	fourier	workforce	level	1.144 (1.012–1.293)	0.032
icu	fourier	workforce	slope change per month	0.993 (0.968–1.019)	0.611
transfer	fourier	covid	level	0.975 (0.934–1.019)	0.257
transfer	fourier	covid	slope change per month	1.001 (0.999–1.003)	0.360
transfer	fourier	recovery	level	1.057 (0.971–1.150)	0.200
transfer	fourier	recovery	slope change per month	1.001 (0.987–1.014)	0.926
transfer	fourier	workforce	level	1.120 (1.051–1.194)	<0.001
transfer	fourier	workforce	slope change per month	1.024 (1.006–1.043)	0.011
admission	fourier	covid	level	1.153 (1.109–1.199)	<0.001
admission	fourier	covid	slope change per month	0.997 (0.996–0.999)	<0.001
admission	fourier	recovery	level	1.074 (1.023–1.129)	0.004
admission	fourier	recovery	slope change per month	0.996 (0.988–1.005)	0.415
admission	fourier	workforce	level	1.152 (1.091–1.217)	<0.001
admission	fourier	workforce	slope change per month	1.046 (1.033–1.059)	<0.001

S10B. Pre-onset trend comparison, preceding 365 days per episode.

Outcome	Comparison	Monthly multiplier (95% CI)	P
visits	COVID pre slope	1.004 (0.995–1.012)	0.407
visits	Workforce pre slope	0.976 (0.968–0.985)	<0.001
visits	pre slope ratio	0.973 (0.965–0.981)	<0.001
icu	COVID pre slope	1.000 (0.991–1.010)	0.924
icu	Workforce pre slope	0.987 (0.978–0.995)	0.002
icu	pre slope ratio	0.986 (0.976–0.997)	0.009
transfer	COVID pre slope	0.994 (0.984–1.004)	0.252
transfer	Workforce pre slope	1.004 (0.995–1.014)	0.391
transfer	pre slope ratio	1.010 (1.001–1.020)	0.030
admission	COVID pre slope	1.001 (0.994–1.008)	0.791
admission	Workforce pre slope	1.004 (0.998–1.011)	0.182
admission	pre slope ratio	1.004 (0.997–1.010)	0.271

S10C. Residual diagnostics.

Outcome	Seasonality	Pearson dispersion	ACF lag 1	ACF lag 7	ACF lag 28
visits	month	1.838	0.393	0.208	0.085
icu	month	1.007	0.026	0.035	0.013
transfer	month	1.092	0.088	0.024	0.018
admission	month	0.866	0.141	0.090	0.050
visits	fourier	1.847	0.401	0.217	0.083
icu	fourier	1.006	0.028	0.036	0.012
transfer	fourier	1.095	0.092	0.028	0.017
admission	fourier	0.868	0.144	0.092	0.052

Full-series N=2,830 daily observations. Level estimates are instantaneous model discontinuities; slope-change estimates multiply the pre-existing monthly trend and are not standalone post-period slopes. Transfer/admission models use daily total visits as the offset. Different pre-onset trends prevent claims of parallel historical trajectories.

Table S11. Alternative baselines and anticipation exclusion.

Sensitivity	Outcome	COVID RR/IRR (95% CI)	Workforce RR/IRR (95% CI)	Ratio (95% CI)
same calendar prior year	Recorded transfer-in	0.92 (0.88–0.97)	1.20 (1.14–1.26)	1.30 (1.21–1.39)
same calendar prior year	Recorded EMS 119 arrival	1.27 (1.23–1.32)	1.04 (1.00–1.09)	0.82 (0.77–0.87)
same calendar prior year	Hospital admission	1.04 (1.01–1.06)	1.14 (1.11–1.16)	1.10 (1.06–1.13)
same calendar prior year	ICU admission	1.00 (0.94–1.06)	1.42 (1.34–1.49)	1.42 (1.31–1.54)
same calendar prior year	Index-visit mortality	1.12 (1.01–1.24)	1.03 (0.93–1.15)	0.92 (0.79–1.07)
same calendar prior year	ED visits/day	0.82 (0.78–0.86)	0.54 (0.51–0.56)	0.65 (0.62–0.70)
same calendar prior year	ED-origin ICU admissions/day	0.93 (0.87–0.98)	0.98 (0.93–1.03)	1.06 (0.97–1.15)
exclude pre onset 14d	Recorded transfer-in	0.96 (0.90–1.02)	1.22 (1.14–1.30)	1.27 (1.19–1.37)
exclude pre onset 14d	Recorded EMS 119 arrival	1.31 (1.25–1.37)	1.09 (1.03–1.15)	0.83 (0.79–0.88)
exclude pre onset 14d	Hospital admission	1.04 (1.00–1.07)	1.15 (1.12–1.19)	1.11 (1.08–1.15)
exclude pre onset 14d	ICU admission	1.00 (0.93–1.07)	1.42 (1.33–1.53)	1.43 (1.32–1.54)
exclude pre onset 14d	Index-visit mortality	1.12 (0.97–1.28)	1.06 (0.92–1.23)	0.95 (0.82–1.11)
exclude pre onset 14d	ED visits/day	0.76 (0.71–0.81)	0.55 (0.52–0.59)	0.73 (0.69–0.78)
exclude pre onset 14d	ED-origin ICU admissions/day	0.91 (0.84–0.98)	1.02 (0.96–1.08)	1.12 (1.04–1.22)

Previous-year baselines: 23 February–3 October 2019 for the COVID episode and 20 February–30 September 2023 for the workforce episode; the corresponding 2020/2024 early windows are unchanged. Each prior-year interval has 223 days versus 224 early days because of leap years; daily counts/offsets handle duration. The ICU-count contrast includes one with this comparator. Anticipation sensitivity excludes 9–22 February 2020 and 6–19 February 2024 from the respective preceding windows.

Table S12. Physiological recording, definition and classification sensitivity.

Abnormal component, n/valid (%)	COVID preceding	COVID early	Workforce preceding	Workforce early
SBP <90	519/21363 (2.4%)	583/18899 (3.1%)	511/20381 (2.5%)	369/12290 (3.0%)
HR >120	1561/21641 (7.2%)	1183/19014 (6.2%)	2952/22405 (13.2%)	1492/13050 (11.4%)
RR <10 or >30	147/21461 (0.7%)	86/18906 (0.5%)	130/21761 (0.6%)	79/12975 (0.6%)
Temperature <35 or >=38	3594/26366 (13.6%)	2140/21266 (10.1%)	2665/22671 (11.8%)	1392/13207 (10.5%)
GCS <=13	1642/26086 (6.3%)	1704/21096 (8.1%)	1582/22294 (7.1%)	1026/12962 (7.9%)

Component thresholds are unchanged from the submitted written definition; the omitted temperature component is now implemented. Different denominators reflect recording availability, not automatically normal values.

Sensitivity	Outcome	N	Ratio (95% CI)	P
all five measured	Recorded physiological abnormality	71931	0.96 (0.90–1.03)	0.257
all five measured	Hospital admission	71913	1.09 (1.05–1.13)	<0.001
all five measured	ICU admission	71913	1.48 (1.36–1.60)	<0.001
all five measured	Index-visit mortality	71913	1.05 (0.87–1.28)	0.609
all five measured	CPR	71913	1.14 (0.77–1.68)	0.529
strict proxy	Recorded physiological abnormality	77503	1.06 (1.00–1.12)	0.034
strict proxy	Hospital admission	77484	1.11 (1.07–1.15)	<0.001
strict proxy	ICU admission	77484	1.47 (1.36–1.59)	<0.001
strict proxy	Index-visit mortality	77484	0.95 (0.82–1.11)	0.548
strict proxy	CPR	77484	1.01 (0.78–1.30)	0.967
four components original	Recorded physiological abnormality	83998	0.82 (0.77–0.89)	<0.001
four components original	Hospital admission	83790	1.12 (1.08–1.16)	<0.001
four components original	ICU admission	83790	1.45 (1.34–1.57)	<0.001
four components original	Index-visit mortality	83790	0.96 (0.83–1.10)	0.529
four components original	CPR	83790	0.95 (0.75–1.22)	0.704
adult only	Recorded physiological abnormality	69577	1.02 (0.96–1.09)	0.496
adult only	Hospital admission	69560	1.07 (1.04–1.11)	<0.001
adult only	ICU admission	69560	1.46 (1.35–1.57)	<0.001
adult only	Index-visit mortality	69560	0.95 (0.81–1.10)	0.480
adult only	CPR	69560	0.97 (0.76–1.23)	0.782
recording completeness adjusted	Recorded physiological abnormality	83623	1.01 (0.96–1.07)	0.697
recording completeness adjusted	Hospital admission	83602	1.12 (1.08–1.16)	<0.001
recording completeness adjusted	ICU admission	83602	1.44 (1.33–1.55)	<0.001
recording completeness adjusted	Index-visit mortality	83602	0.97 (0.84–1.11)	0.643
recording completeness adjusted	CPR	83602	0.99 (0.80–1.23)	0.943

S12B. Additional source-classification sensitivities.

Sensitivity	Outcome	Ratio (95% CI)	P
complete route transport clinical visits	Recorded transfer-in	1.28 (1.20–1.38)	<0.001
complete route transport clinical visits	Recorded EMS 119 arrival	0.84 (0.80–0.89)	<0.001
complete route transport clinical visits	Hospital admission	1.12 (1.08–1.15)	<0.001
complete route transport clinical visits	ICU admission	1.44 (1.33–1.56)	<0.001
complete route transport clinical visits	Index-visit mortality	0.95 (0.82–1.11)	0.524
exclude DOA	Index-visit mortality	0.99 (0.85–1.16)	0.942

Table S13. Recorded care intervals and outcome coverage.

Interval: n/eligible; median [IQR]	COVID preceding	COVID early	Workforce preceding	Workforce early
ed los hours	26,480/26,481; 3.80 [2.45–5.50]	21,396/21,396; 3.83 [2.60–5.49]	22,779/22,780; 3.92 [2.63–5.82]	13,341/13,341; 3.00 [2.10–4.20]
ed los admitted	10,127/10,128; 4.88 [3.50–6.88]	9,184/9,184; 4.53 [3.25–6.53]	9,560/9,561; 4.79 [3.40–7.20]	7,327/7,327; 3.13 [2.27–4.35]
ed los not admitted	16,353/16,353; 3.18 [2.05–4.62]	12,212/12,212; 3.37 [2.20–4.80]	13,219/13,219; 3.33 [2.22–4.90]	6,014/6,014; 2.82 [1.88–4.02]
order to exit hours	9,783/10,128; 1.29 [0.68–2.62]	8,906/9,184; 1.24 [0.67–2.44]	9,334/9,561; 1.60 [0.84–3.29]	7,107/7,327; 1.00 [0.55–1.72]
icu calendar days	1,911/2,113; 3.00 [2.00–7.00]	1,787/1,964; 3.00 [1.00–7.00]	1,942/2,070; 4.00 [2.00–8.00]	1,964/2,166; 3.00 [1.00–7.00]
Coverage/audit item	COVID preceding	COVID early	Workforce preceding	Workforce early
ed los valid	26480	21396	22779	13341
admitted order present	10123	9183	9559	7327
admitted order sequence valid	9783	8906	9334	7107
icu outcome	2113	1964	2070	2166
icu interval recorded	1911	1787	1943	1985
icu interval valid	1911	1787	1942	1964
hospital discharge missing	0	1	2	22
death reconstruction disagreements	0	0	0	0

ED/order-to-departure intervals are hours; ICU intervals are calendar-day differences only. Date strings were YYYYMMDD, so hour-precision ICU LOS was not invented. Bed assignment is not actual transfer. Only valid completed intervals are summarised; no missing/censored interval is assigned zero. Shorter intervals do not establish efficiency or staffing adequacy.

Mortality sensitivity	COVID aRR (95% CI)	Workforce aRR (95% CI)	Ratio (95% CI)
Exclude unresolved admitted episodes	1.11 (0.97–1.27)	1.06 (0.92–1.22)	0.95 (0.82–1.11)

Table S14. Transparent changes from submitted analysis.

Topic	Submitted limitation/discrepancy	Revision and consequence
Age units	1,219 primary-window month-coded infant ages were converted to missing.	Parse 0m–11m as months/12; no age imputation. Age distributions and all affected models recomputed.
Physiology definition	Written definition included temperature, but the archived composite used only SBP/HR/RR/GCS.	Implement all five stated components; retain four-component results only as labelled sensitivity. The previous comparative physiological claim is withdrawn.
Partial/all-missing physiology	Sentinels and partial recording were not sufficiently documented.	Apply explicit validity screens; all-unavailable composite is missing; distinguish no recorded abnormality from complete normality.
Correlation	Patient-only clustering did not address same-day common conditions.	Primary patient/day two-way variance; separate daily-score HAC sensitivities.
Daily-volume specification	Table S1 used Fourier while the principal count model used categorical month.	Single categorical-month duration pipeline. Both specifications shown separately in Table S3; actual-calendar lags used throughout.
Multiplicity	No single confirmatory main endpoint; main interaction tests nominal.	Exploratory domain comparisons; review-added ten-test BH family, separate nine-diagnosis family.
Time endpoints	ICU admissions/day could be mistaken for actual-day census; ICU timestamps assumed.	Label ED-origin counts by ED arrival date; report ICU intervals only at exported calendar-date precision.
Interpretation	Pressure/workload and physiological differences were stronger than supported.	Use utilisation/flow wording; report less-conditioned mortality, nonparallel pretrends and baseline-sensitive absolute ICU contrast.

All corrections and reviewer-requested analyses were performed during revision, not retrospectively labelled as prespecified. Counts of visits and the original admission/ICU/death/CPR events are unchanged; covariate availability, some case-mix statistics, coefficients and uncertainty estimates changed. Model equations and fixed aggregate outputs accompany the analysis code.

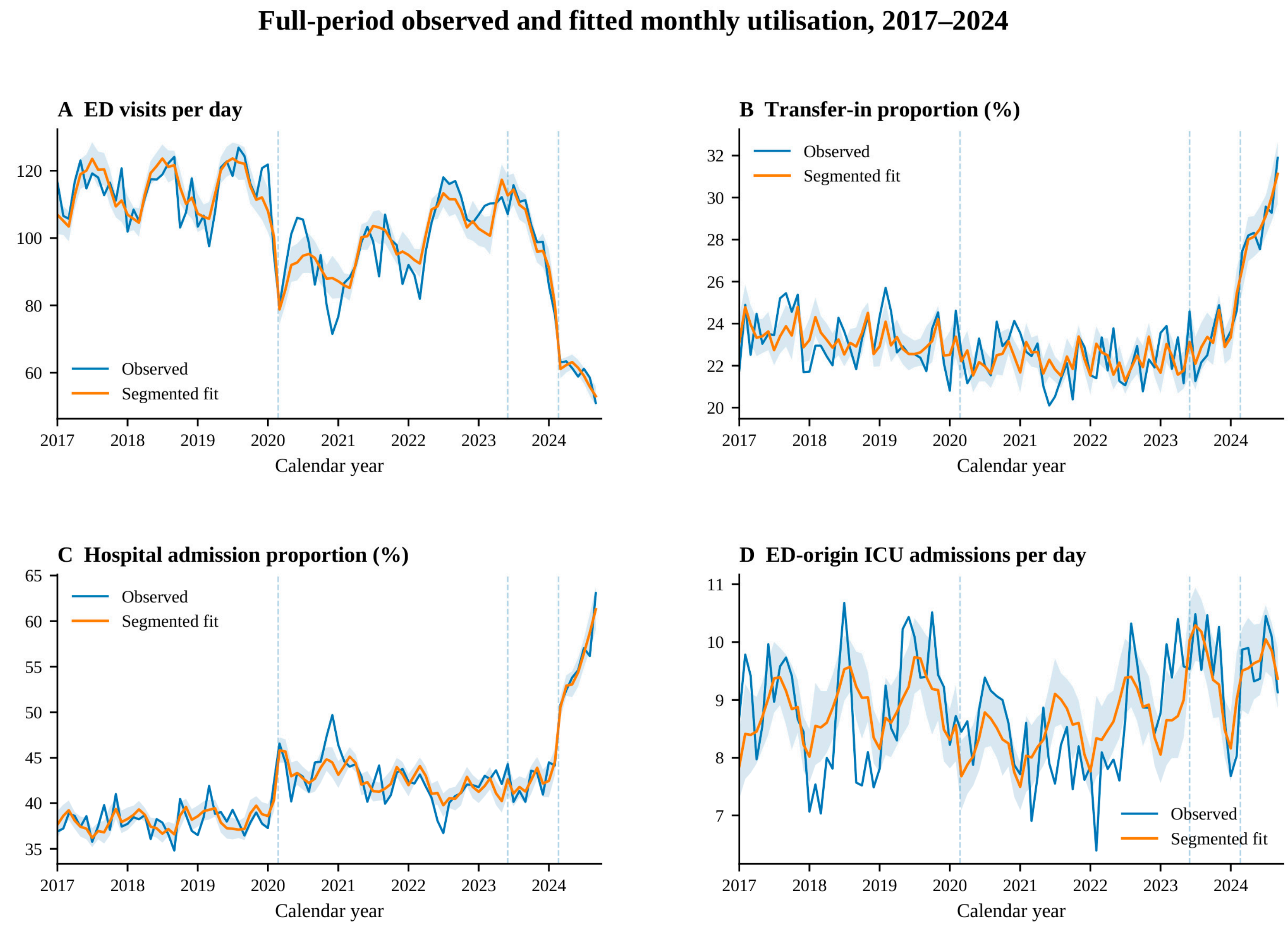

Figure S1. Monthly observed utilisation and fitted means from the full-period segmented daily analyses, 2017–September 2024. Bands are pointwise 95% confidence intervals for fitted monthly means, not prediction intervals. Vertical lines mark 23 February 2020, 1 June 2023 and 20 February 2024. The model includes linear time and level/slope changes at these dates, categorical month, day of week and HAC(28) variance. The unexposed counterfactual is not identified; fitted curves do not establish causal effects.

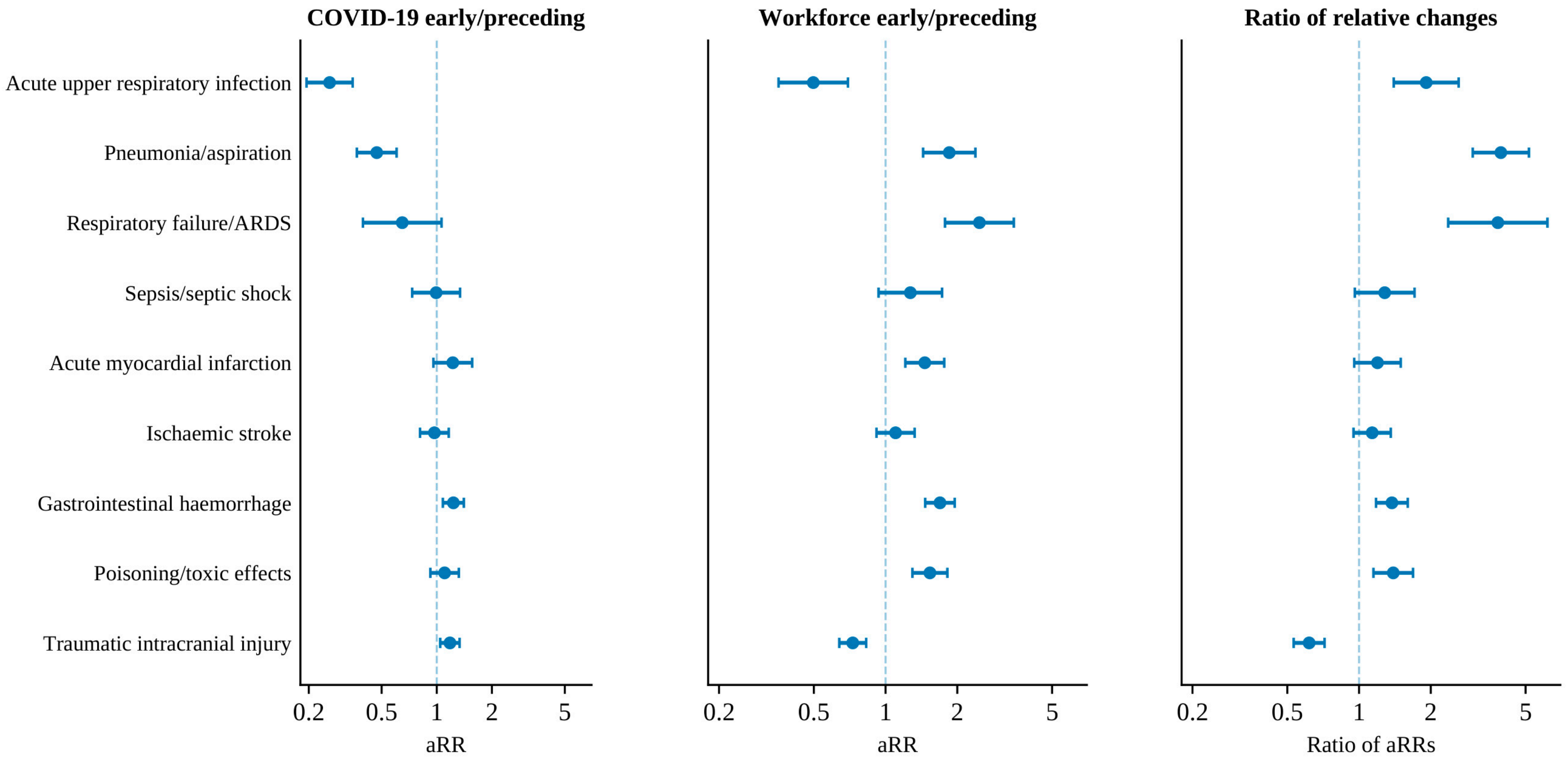

Figure S2. Full nine-group diagnostic-composition comparisons. Points show adjusted rate ratios or their ratios on logarithmic scales; bars show 95% confidence intervals from calendar-lag HAC(7). Daily visit counts supply the offset. Sepsis is included in the nine-group interaction FDR family. Estimates are based on the same fixed diagnostic codebook as Table S5.

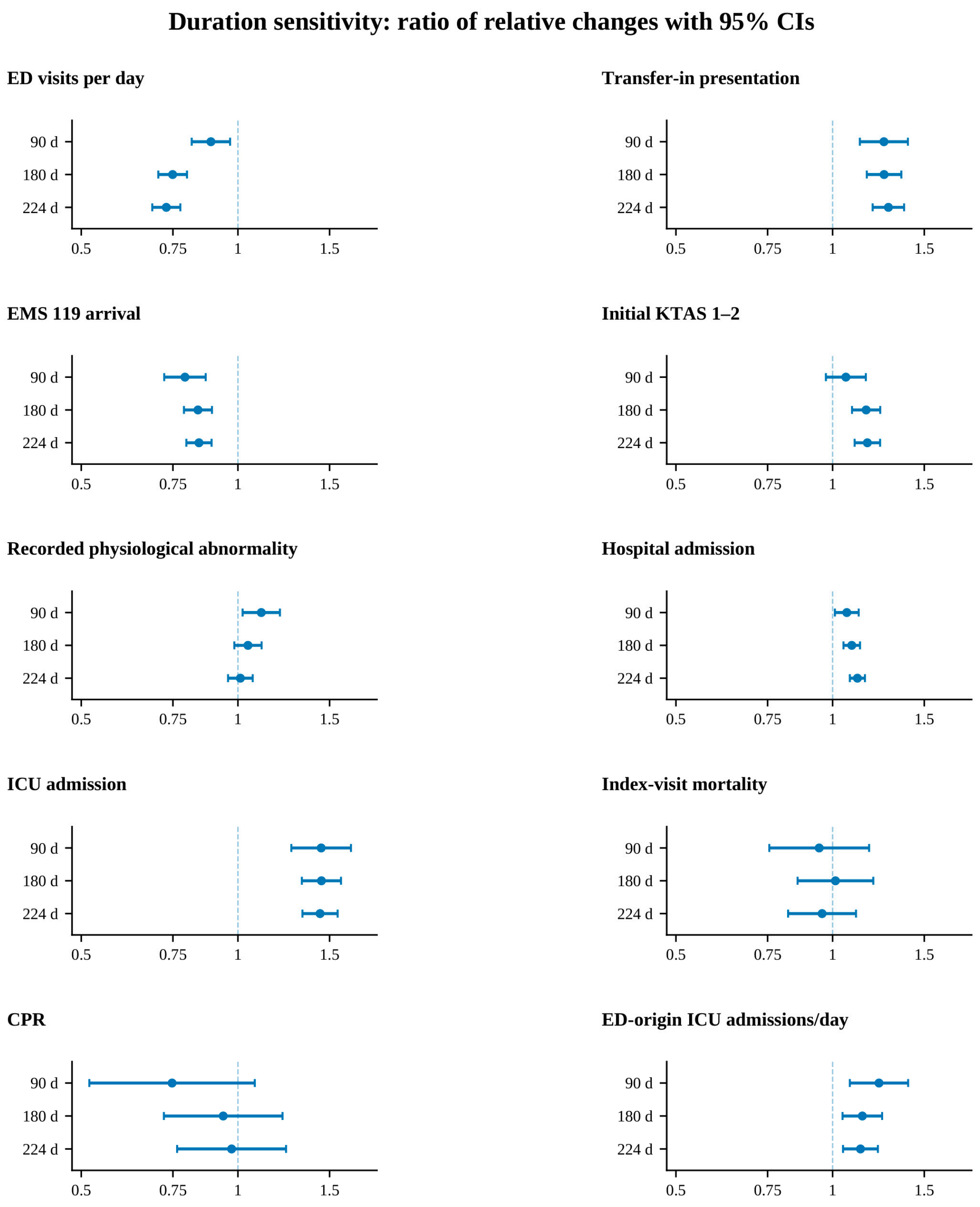

Figure S3. Window-length sensitivity for all ten main outcomes. Each estimate compares the workforce early/preceding relative change with the COVID-19 early/preceding change. Error bars show pointwise 95% confidence intervals on a logarithmic scale. All durations use the same categorical-month specification and corrected variables. Patient/day clustering is used for binary outcomes and calendar-lag HAC(7) for daily counts. The physiological contrast and short-window KTAS contrast are not uniformly robust.
